# Cell polarity: having and making sense of direction—on the evolutionary significance of the primary cilium/centrosome organ in Metazoa

**DOI:** 10.1098/rsob.180052

**Published:** 2018-08-01

**Authors:** Michel Bornens

**Affiliations:** Institut Curie, PSL Research University, CNRS - UMR 144, 75005 Paris, France

**Keywords:** centrosome, primary cellium, evolution, cell polarity, individuality, sensorimotricity

## Abstract

Cell-autonomous polarity in Metazoans is evolutionarily conserved. I assume that permanent polarity in unicellular eukaryotes is required for cell motion and sensory reception, integration of these two activities being an evolutionarily constrained function. Metazoans are unique in making cohesive multicellular organisms through complete cell divisions. They evolved a primary cilium/centrosome (PC/C) organ, ensuring similar functions to the basal body/flagellum of unicellular eukaryotes, but in different cells, or in the same cell at different moments. The possibility that this innovation contributed to the evolution of individuality, in being instrumental in the early specification of the germ line during development, is further discussed. Then, using the example of highly regenerative organisms like planarians, which have lost PC/C organ in dividing cells, I discuss the possibility that part of the remodelling necessary to reach a new higher-level unit of selection in multi-cellular organisms has been triggered by conflicts among individual cell polarities to reach an organismic polarity. Finally, I briefly consider organisms with a sensorimotor organ like the brain that requires exceedingly elongated polarized cells for its activity. I conclude that beyond critical consequences for embryo development, the conservation of cell-autonomous polarity in Metazoans had far-reaching implications for the evolution of individuality.

## 1. Introduction

Cell polarity (i.e. vectorial activity supported by asymmetric cell organization, and maintained by appropriate signalling) is an essential feature of animal cells. It is critical for embryo development. Early cell signalling depends upon stem cell polarity and pre-patterning of polarized cells [1,2], while gastrulation, organogenesis and tissue activity rest on the polarized activity of individual cells and on directed cell migration. Cell fate determination in most tissues is rooted in asymmetry of intrinsic polarity cues during the division of stem cells [3–7]. A considerable body of knowledge has been accumulated over the years on molecular regulations of plasma membrane polarity in relation with extrinsic and intrinsic cues [8–16]. In unicellular organisms, cell polarity cues are always linked with cell division. In animals, during tissue growth, the polarized activity of cells is preserved by the controlled orientation of division axis, allowing proper transmission of mother cell polarity to daughter cells [17–22]. Terminally differentiated cells keep a polarized organization in most lineages in vertebrates. They can however relax to a symmetrical organization in some lineages. This is the case for example during skeletal muscle differentiation in vertebrates, where myotubes, although anisotropic, have no front and rear ends. It is noteworthy that impaired or unstable cell polarity is a hallmark of malignant transformation, as this apparently has the potential to trigger tissue destabilization [23].

In this Perspective, I want to investigate whether a comprehensive understanding of the significance of permanent symmetry breaking in animal cells is possible. This is done in an evolutionary perspective, as it is the condition for clarifying critical issues in cell biology [24]. It is not an attempt to propose an additional scenario for animal evolution that would complete, or correct, the current views on this matter. Nor is it an attempt to address theoretical questions on biological evolution. Instead, it is a survey of established experimental data from distinct and usually disconnected domains. The aim, in front of the bewildering richness of phenomena and of ‘the twin difficulties of scale and complexity’ [25], is to look for a unified description, at the cell level, that could shed light on all these domains. I will show that all experimental data support, with reasonable assumptions in some cases, the contention that cell-autonomous polarity is a critical cell feature connecting all these domains. For the sake of brevity, I will not go beyond a rapid survey of the different domains, and I will often refer the reader to reviews for a more comprehensive vision in each of them.

The main conclusion—a provisional conclusion indeed—is that cell-autonomous polarity has a pivotal role not only at all levels of animal living organization, but also for the evolution of individuality^1^ in Metazoa.

## 2. Why are unicellular eukaryotes polarized?

**Figure 1.**
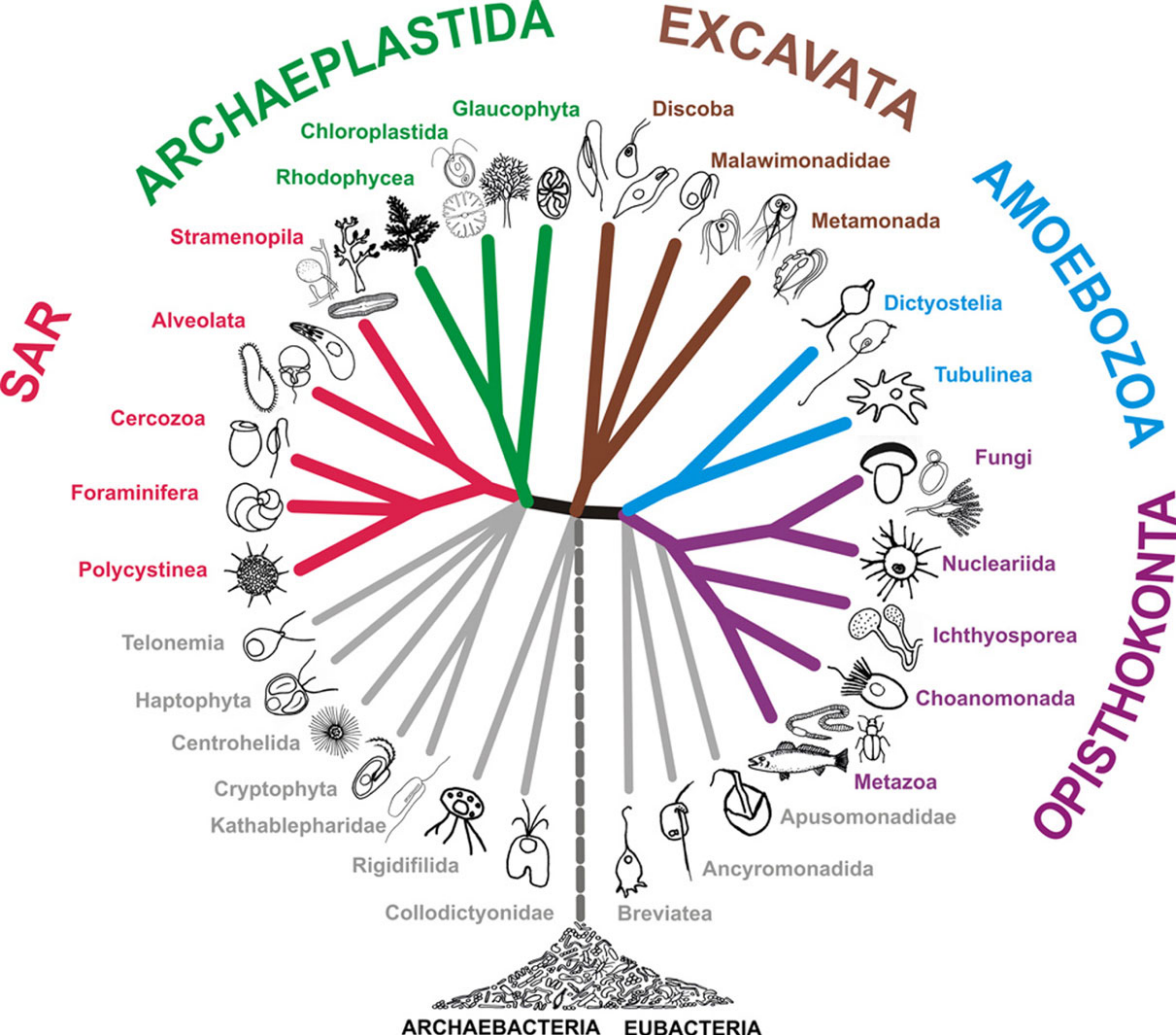
Eukaryote phylogeny reflecting the classification presented in Adl *et al*. [27].

The overwhelming majority of lineages in eukaryotes are exclusively, or almost exclusively, unicellular organisms [27] (figure 1). It is established beyond any doubt, that cell polarity in Metazoans is, for a large part, evolutionary conserved rather than a derived character, placing the origin of cell polarity in the unicellular ancestors (see [28,29]).

The ability to move is critical for microorganisms which experience permanent changes in their habitats. An insightful and unified vision on cell movements—a very rich field of phenomena—can be found in ref. [30]. Spatial asymmetry is a general property of unicellular organisms. Rod-shaped bacteria, for example, are asymmetric by construction: although they have very different ways to set up functional polarity [31], the two poles of a rod-shape bacterium are different, the new one having being made in the preceeding division, the old one in an older division. This generational asymmetry, and the integration of cell cycle regulators with polar maturation, is a way to set bacterial polarity [31].

Owing to the respective cell sizes, there are indeed considerable differences in the strategies to move and to respond to chemical gradients between unicellular eukaryotes and Bacteria or Archaea, on which Brownian movement has a strong impact [30,32]. Note that unicellular eukaryotes differ from bacteria not only in terms of size, compartmentalization and metabolism, but also in their mode of feeding [33,34]. Many unicellular eukaryotes are phagotrophic: they feed by engulfing food—either whole cells, like bacteria or other unicellular eukaryotes, or particles—and ingest them in a phagocytic vacuole, whereas prokaryotes, with their size in the micrometre range (i.e. in the range of a phagocytic vacuole), are osmotrophic or phototrophic. The positioning of the phagocytic machinery in unicellular eukaryotes is not random, and most often coincides with the polarity axis. As a matter of fact, flagella or cilia beating is quite often used to favour feeding. Apparently, sensation, locomotion and feeding were not selected independently. Classically, a flagellum allows locomotion but its beating creates potent and directed currents in the surrounding medium that are used for capturing food. All sorts of solutions were selected, from the flagellar pocket of *Trypanosoma* [35] or the gullet of *Paramecium* [36] to the surprising case of the biflagellate chrysophicean *Epipyxis pulchra*, which chooses its prey after capturing them with its two different flagella, and then assembles a basal body-associated microtubule bundle forming a buckle under the plasma membrane to create a transient engulfing structure, whenever a good prey is captured [37]. Feeding–swimming trade-off is conserved in larval stages of many multicellular marine organisms (see §4.3).

Therefore, cell sensorimotricity appears as an evolutionary selected functional module. As they experience permanent changes in their habitats, the ability for eukaryotic microorganisms to move, either for feeding or for fleeing from unfavourable spots, is critical. Any improvement in their ability to sense food, or predators and toxic environment, while moving, would bring a selective advantage (see [28,29]).

As addressed in §2.3, the flagellum/cilium of eukaryotes is indeed also a sensory organelle [33]. This very brief overview indicates that the direction of membrane traffic in unicellular eukaryotes is set by the positioning of the phagocytic machinery and thus, most often, coincides with that of the flagellar apparatus. This could be at the origin of the polarized membrane traffic in animal cells [38].

### 2.1. Flagellum-dependent cell locomotion and feeding

It is currently admitted that the most ancient common eukaryote ancestor had a quite complex microtubule cytoskeleton, similar to that of extant members of the super-clade Excavata. It was probably a phagotrophic biflagellate forming a ventral feeding groove, with a posterior basal body assembling a flagellum beating within the ventral groove to facilitate prey capture [39–41] and an anterior basal body assembling a flagellum for gliding locomotion. Gliding is apparently a very ancient strategy to move on a surface in spite of the high viscous drag for this type of locomotion [42]. For a detailed analysis of the evolution of cilia, see [43,44].

#### 2.1.1. The basal body

**Figure 2.**
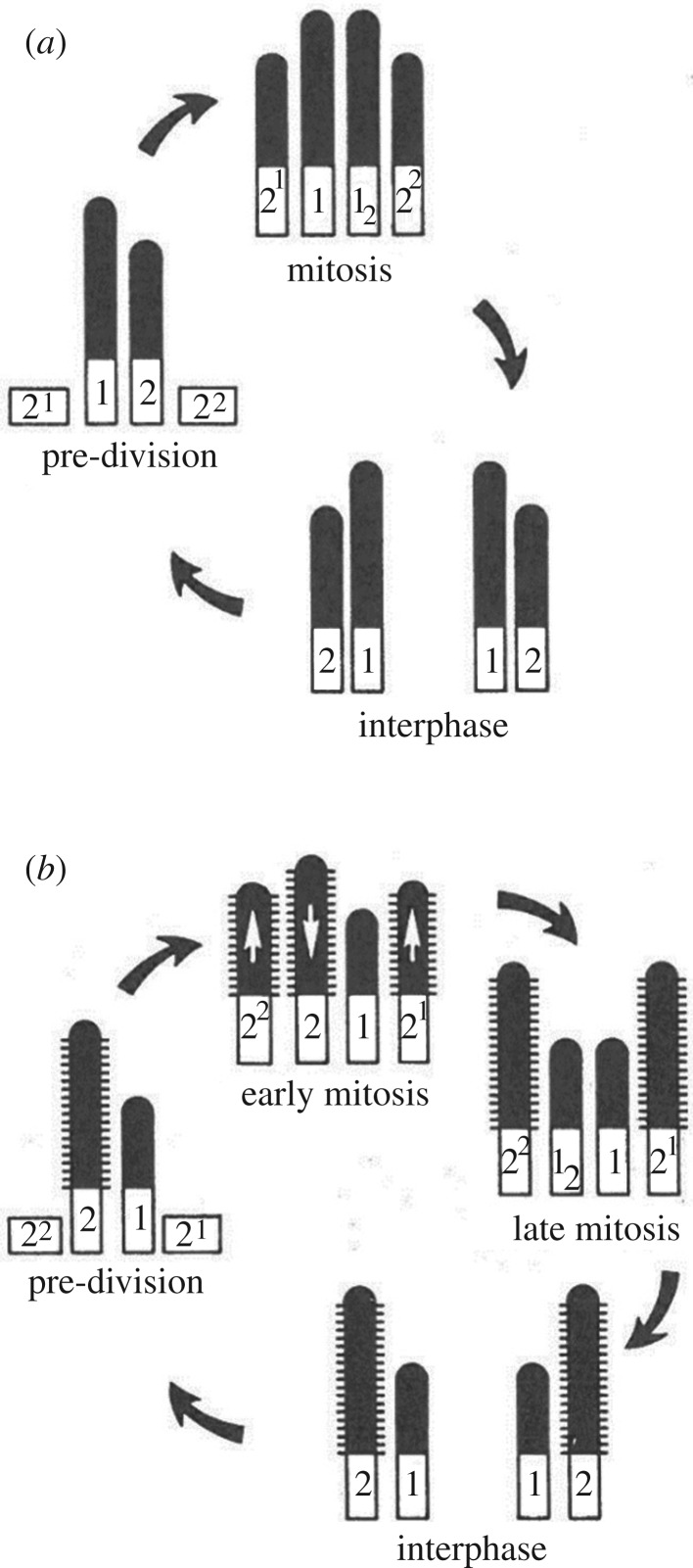
Two different flagellar development cycles in unicellular algae: (*a*) *Nephroselmis olivacea* and (*b*) *E. pulchra* (adapted from Beech *et al*. [48]).

**Figure 3.**
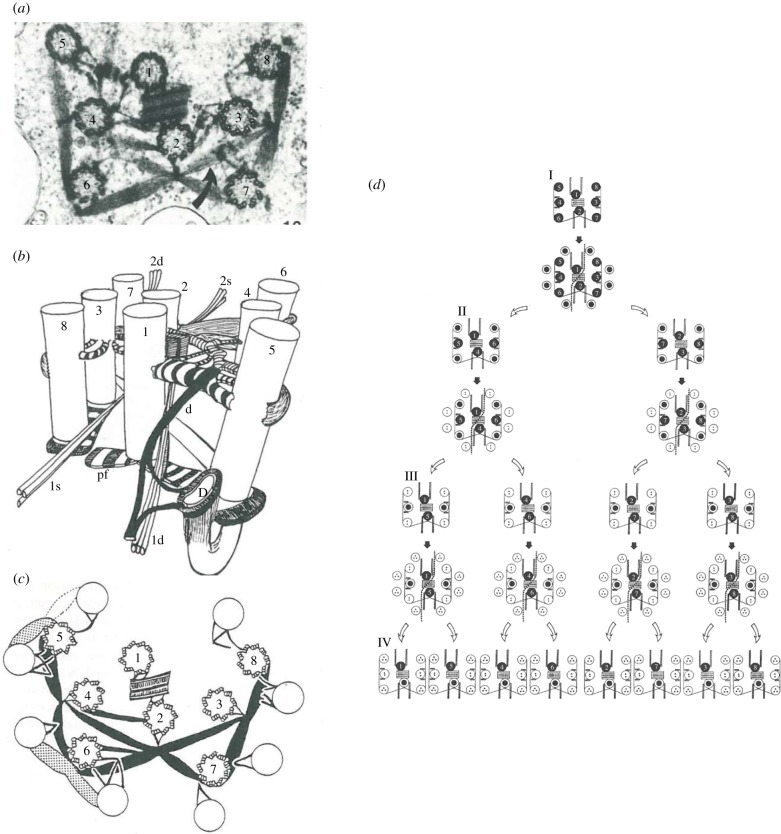
(*a,b*) The flagellar apparatus of *Pyramimonas octopus*, (*c*) a stage in flagellar duplication and (*d*) the flagellar shift in subsequent cell generations. There are at least 60 connecting fibres, including the central synostosome (possibly contractile for basal bodies reorientation), six (possibly huit) rhizoplasts connecting the flagellar apparatus to the nucleus and the chloroplast, and four microtubular flagellar roots. During basal body duplication, most if not all connecting fibres break down and are reformed when the basal bodies shift to new positions. Three sequential generations are necessary until the eight original basal bodies all reach the definitive position. Whether the synostosome is retained, shared, or whether two new synostosomes are assembled is not known (adapted from Moestrup [49]).

Most significantly, the 0.2 µm diameter, ninefold radially symmetrical basal body, made of long-lived microtubules, which templates the axoneme and nucleates a parietal pellicle of microtubules in swimming unicellular organisms, is a derived character of eukaryotes [45]. Moreover, the unique stability of these structures is conserved through evolution: the sperm basal bodies which form the centrioles in the egg in the worm *Caenorhabditis elegans* are apparently the only cellular structures that can be passed virtually unchanged from one cell cycle to the next through many cell generations [46]. In addition, although a new basal body can assemble *de novo*, it usually reproduces according to a precise mechanism of conservative duplication [47]. This imposes a control on the number and position of new basal bodies with respect to the parental ones, and sets a generational asymmetry between the old and the new basal body. This generational asymmetry, easily observed in biflagellates that undertake flagella transformation—they grow different flagella according to the age of the basal bodies [48] (figure 2)—has important implications for the reproduction of the whole-cell morphology (see §2.1.4). Flagellar transformation might require several cell division cycles to reach full basal body maturation, when the number of flagella is higher than two. For example, the unicellular green algae *Pyramimonas octopus* has eight flagella, each of them having a hierarchical position in terms of maturation, while the oldest has reached a definitive position, aside the centrally located synostome [49] (figure 3). It takes three cell cycles for all the seven basal bodies from the great-grandmother cell to progressively reach the position near the synostome, corresponding to full maturation in each of the seven great-granddaughter cells, the eighth great-granddaughter cell having the oldest basal body at the correct position to start with (for a physical approach of the positioning of the basal bodies in these types of multiflagellate algae, see [50]). A similar transformation among eight flagella can be observed in the diplomonad *Giardia intestinalis* [51].

The most common reproduction of basal bodies, through an apparently conservative duplication mechanism, results in their spatially restricted continuity. Their generational maturation sets a lineage which defines an arrow of time.

#### 2.1.2. The basal body connections

The eukaryotic basal body–flagellum displays specific features. First, the polarized positioning of the flagella apparatus, which may contain one, two or several flagella, at the surface of the cell body in many unicellular eukaryotes, is firmly associated by a structural connection with the nucleus, the so-called nuclear-basal body connector, or rhizoplast, in unicellular algae [52–54]. Such a physical connection is often conspicuous, like in the Amoebozoa *Physarum polycephalum* [55] or *Dictyostelium discoideum,* which displays a centrosome without centrioles [56]. It is conserved, but more diffuse, in animal cells [57]. It could be instrumental in the necessary coordination between the duplication of DNA and that of basal bodies during each cell division cycle [28] (see also §4.1). However, it is not always observed; in kinetoplastidae, for example, the basal body is not connected to the nucleus but to the kinetoplast instead [58,59], and in ciliates a completely different strategy is used (see §2.4), indicating that other ways to coordinate karyokinesis and cytokinesis can exist. In animal cells, the association of the centrosome to the nucleus and that of primary cilium to the plasma membrane, could be a modified version of the ancestral connector between nucleus and plasma membrane (see §§3.1.3 and 3.2). Second, basal bodies are associated with three to four different MT roots which play a key role for cell shape. They can be recognized, in spite of their evolution, among different unicellular eukaryotes, suggesting that the ancestral MT cytoskeleton was as complex as that of extant Excavata [38–40]. Third, basal bodies are indeed inserted, through nine radial distal appendages, in the plasma membrane to grow flagella.

Although with variations among the different unicellular eukaryotes, the basic requirements for a permanent and direct interfacing between plasma membrane and a MT-based cytoskeletal structure, with the setting of a diffusion barrier, are similar and ensured by conserved gene products.

Finally, basal bodies, or centrioles, are the only structures in which microtubule triplets are present, even if these triplets can extend only on the proximal part of the centrioles in human cells [60]. The precise function of these triplets is not known, although they appear necessary for radial connections around the basal body [61]. It has been shown in several unicellular eukaryotes that the rare δ- and ε-tubulins are necessary for triplets assembly [62,63]. A recent report has shown that this is also the case in human cells [64], and that the absence of triplet formation precludes the formation of the distal part of centrioles, as judged by the absence of recruitment of distal proteins that are necessary for its assembly [65], leading to unstable centrioles that cannot be inherited from one cell cycle to the next.

#### 2.1.3. The eukaryote flagellum

The eukaryote flagellum is a genuine intra-cellular compartment, with a diffusion barrier at the base, whereas prokaryotic flagella are polymers projecting outside the cell body [32]. The overall structure and diameter of the basal body/axoneme, considerably larger than that of prokaryotes flagella, are remarkably conserved in eukaryotes [43], with the so-called (9 × 2 + 2) pattern. The complex structure of the axoneme is correlated by more than 600 constitutive proteins [66], which all have evolved in their primary structure among species, in spite of the striking conservation of the overall flagellum structure, from unicellular organisms to the human species. Constraints imposing such an evolutionary invariance are probably linked to the preservation of the flagellar beating. The complex structure of the eukaryotic flagellum contrast bluntly with that of Bacteria or Archaea flagella, which correspond to polymers of identical monomer subunits. It produces a beating movement against the water by intracellular dynein-dependent sliding of adjacent doublets with respect to each other, whereas extracellular curly bacterial or archaeal flagella, which look alike but are of quite different origin [67,68], produce movement by rotating.

The conserved axoneme structure is extensively distributed in uni- and multicellular eukaryotes. Its evolutionary success is considerable and cannot be over-estimated. We have no real clue about how it first appeared.

#### 2.1.4. Flagellum and cell division: the distinct ways of maintaining cell polarity

For unicellular or multicellular organisms, cell division requires a coordination between karyokinesis and cytokinesis, which can be achieved in very distinct ways. For example, when cells keep swimming during division, this imposes a complex division process: uniflagellate trypanosomes, which belong to the Excavata super-group, reproduce their complex and polarized cortical organization by starting cell division with the duplication of the basal body/axoneme, localized at the most anterior part of the cell cortex [69]. The separation of the duplicated basal bodies proceeds without apparent connection with karyokinesis, but rather with the segregation of kinetoplast DNA. The doubling of microtubule number in the parietal pellicle, necessary to support the shape of the two daughter cells, takes place according to a very precise process mixing old and new microtubules [70]. Cell division terminates by the separation of the mother and the daughter cell at the tip of the old and the new flagellum, thus allowing a precise transmission of the whole polar organization of the mother cell to the daughter cell. In the meantime, the lateral attachment of the new flagellum to the cell body is a key morphogenetic structure [71]. Another example is provided by a group of asymmetric unicellular biflagellate algae common in marine and freshwater habitats, the cryptomonads. Cell division involves a complete resetting of cell polarity, the so-called ‘polar reversal’ [72], where the posterior tail-like region of each daughter cell develops from the anterior part of the mother cell.

In other biflagellate algae like *Chamydomonas reinhardtii*, flagella are resorbed during cell division, and basal body reorientation apparently suffices to maintain cell asymmetry. The inherent asymmetry between the old and the new basal body has a critical role to preserve overall cell handedness: the two sides of *C. reinhardtii* for example are largely symmetric, but can be distinguished by the location of the single eyespot. The asymmetry of the basal body pair appears to control the invariant handedness of the eyespot position, and mating structure position [73,74]. The mating structure itself forms upon the intimate adhesion between flagella from the two mating partners, and the asymmetric flagellar apparatus in each cell ensures the mating process can take place successfully.

Thus, in many unicellular eukaryotes, there is a trade-off between motility and division: cells either move or divide. They shed or resorb their flagellum at the onset of mitosis and do not swim during the whole division process [75]. This is classically interpreted as meaning that motility and division compete for the same machinery (but see §3.3.2).

Cell-cycle-dependent behaviour is apparently conserved in animal organisms, where cells resorb they primary cilium when they enter a new cell division cycle. In addition, they stop migrating and round up transiently during mitosis [76].

Interestingly, the very short division cycle of some uniflagellate bacteria, like *Caulobacter crescentus,* is reminiscent of the trade-off between motility and division that is observed in eukaryotic cells for preserving cell polarity [77]: asymmetric division leads to swimming and immobile daughter cells, an apparently efficient—and prudent—strategy to explore new environments. Remarkably, the onset of DNA replication is not synchronous in the two daughter cells. It is delayed in the swimming cell, until the cell becomes immotile, replacing its polarized flagellum by a stalk. In the immobile daughter cell that inherited the polarized stalk of the mother cell, DNA replication starts without delay after complete division. What sort of trade-off could explain the incompatibility between cell moving and replicating DNA observed in that case is not known.

#### 2.1.5. Generational asymmetry of basal bodies and cell generational asymmetry

The tight temporal coupling between the reproduction of the genome and that of the flagellar apparatus prepares their mechanistic coupling during karyokinesis. The success of cell division itself depends on the connections of the spindle poles with the basal bodies, which in all cases maintain also a connection with the cell cortex. The connection of the spindle poles with the basal bodies can be indirect*,* like in the extra nuclear pleuromitosis of *Trichomonas,* through specific structures (see [78] and references therein).

The two mitotic spindle poles always display a generational asymmetry. Importantly, this asymmetry may not be strictly limited to basal bodies themselves; it may encompass other compartments, like plasma or internal membranes, to which duplicated basal bodies become asymmetrically connected in one way or another during their duplication. This is maintained in animal cells, in which examples have been documented [79,80]. In some cases, this could correspond to a whole cellular module such as the apicosome recently described in human pluripotent stem cells, which is asymmetrically inherited after mitosis [81]. In all cases, the generational asymmetry of the basal bodies would thus ensure a structural continuity through cell division, reminiscent of the cortical inheritance observed in ciliates (see §2.4). Asymmetry is a critical feature of animal stem cells, in which divisions produce daughter cells with different fates [7]. This constitutive asymmetry of the poles is structurally cryptic in proliferative divisions of most animal somatic cells. It may, however, play an important role for preserving cellular metabolism and long-lived progeny: asymmetric segregation of proteins destined to degradation as has been observed in cultured cells [82], as well as that of aggresomes *in vivo,* where their segregation occurs with a fixed polarity during development [83].

After successful karyokinesis, the dividing cell has still to pass through risky processes: cytokinesis failure is not infrequent in animal cells, and this can precede malignant transformation. The two new nuclei have to be correctly positioned and connected to cytoplasmic structures from each presumptive daughter cell, new origins of DNA replication have to be set, and vectorial activity has to be rapidly reset in the two presumptive daughter cells after telophase, before abscission. Pre-assembled basal bodies, with associated structures according to their generational ranking and their orientation, are critical for cell polarity resetting. This strategy has been apparently successfully conserved by animal cells in tissue.

In conclusion, whatever the topological scenario of cell division (see §3.3.2), as symmetric genome transmission proceeds, asymmetric transmission of pre-assembled intrinsic polarity cues proceeds with it, the condition for the two post-mitotic cells to rapidly reset vectorial activity while maintaining a generational continuity between them.

### 2.2. Cell migration

Unicellular filopodiated eukaryotes display other ways to move than flagellum-dependent swimming or gliding. The last common eukaryotic ancestor was probably able, depending on the life cycle and of the environment, to switch from swimming to amoeboid motion. Two other unicellular lineages, in addition to choanoflagellates, are most closely related to animals, namely the filopodiated filastereans, and the ichthyosporeans [84,85]. Ichthyosporeans reproduce through coenocytes and disperse as flagellate or amoeboid forms. The gene content of the unicellular ancestor of animals thus display a surprisingly rich repertoire of genes present in modern animals. In addition, recent evidence has been provided that choanoflagellates themselves are able to have amoeboid movements, while keeping their flagellum [86]. Such a versatility in the mode of locomotion seems actually a surprisingly common feature. It has been also observed in cells from fungi: the ‘lower’ fungi chytrids have flagellate zoospores that can also move with an amoeboid movement while conserving their flagellum [87]. The plasmodial amoebozoan *P. polycephalum* can also show a switch depending on the environment, between flagellated cells, in a wet environment, and amoeba, in a dry environment [88]. Another type of versatility in cell motion, observed in extant amoebo-flagellates, like *Naegleria gruberi*, belonging to the Excavata super-clade [89], shows that the transition between the two modes can be very rapid. This unicellular organism lacks any cytoplasmic tubulin-based polymers, having only an intra-nuclear spindle during mitosis. It can switch in 1 h from an amoeba form to a swarm cell with a highly packed cortical pellicle of microtubules, organized about two basal bodies and two flagella [47]. Interestingly, cortical actin participates in this transition [90].

Most often, with rare exceptions such as sperm cells from nematodes [91], cell migration rests on an actin-based network, which demonstrates a considerable plasticity in its organization and composition, depending on the cell type and on the environment. It can show spontaneous symmetry breaking, maintained by positive feedback loops. The possibility that such mechanisms can lead by itself to self-polarization and stable axis of migration has been demonstrated using beads in an *in vitro* system mimicking the *Listeria* movement within mammalian cells [92,93]. It has also been directly provoked on discoid stationary lamellar fragments of epidermal fish keratocytes, by mechanically imposing a rear edge, leading to stable migration of the fragments in the opposite direction, as the retrograde actin flow from the periphery of fragments was no longer symmetrical [94].

Indeed, an evolution of the actin system, and of the cytoskeleton as a whole, has taken place between uni- and multicellular organisms [95,96]. There are conspicuous differences between the migration of unicellular organisms like the rhizopode *Amoeba proteus,* which displays a dramatic change in shape (https://www.youtube.com/watch?v=mv6Ehv06mXY*;)* without obvious organizational stable polarity [97], and that of cells from animal organisms, which keep a more constant and polarized shape while migrating (https://www.youtube.com/watch?v=I_xh-bkiv_c). In the first case, an anterograde movement of the fluid endoplasm is opposed to its ecto-cytoplasmic gel, while in the second case, a retrograde actin flow results from actin polymerization at the front membrane, which can be used in an adhesion-dependent or independent motility [98].

In most animal cells, the actin flow which responds to extrinsic environmental cues acts on intrinsic polarity cues to re-orient them [99].

Adhesion on a surface can also be observed in wall-less bacteria like mycoplasms, which adhere strongly on the surface of eukaryote cells. Interestingly, *Mycoplasma pneumonia* has an apparently conservative, template-driven mode of duplication of the terminal organelle, or attachment organelle, which has a polar location and whose assembly is coordinated with the cell cycle [100].

### 2.3. Sensorimotricity, a cell evolutionarily constrained functional module

Although long being neglected compared with motion, the ability of cilia/flagella from unicellular organisms to be sensory devices, just like primary cilia of Metazoa, is supported by many data [33,101]. Sensation of the environment and motion must be integrated at the cell level. A blind motility (i.e. a cell motility without some sort of feedback on motor activity), according to stimuli encountered by the moving cell in its environment, would probably be very inefficient and of poor survival value. Any evolutionary gain in motion efficiency would be probably detrimental if not accompanied by a gain in signalling efficiency (see [28]). The cilium/flagellum compartment of eukaryotes has a great surface/volume ratio and all its surface in contact with the extracellular medium. Thanks to a diffusion barrier at the base of the flagellum, sensory receptors can be specifically concentrated on the ciliary membrane [102–104]*.* Unicellular organisms are able to monitor and to respond to all sorts of physical and chemical stimuli. Mechanisms for coupling motion and sensation have been documented at the molecular level in impressive details in bacteria [105–108] although a lot is still missing [109]. They are quite different from mechanisms existing in eukaryotes, which are also far from being described in a comprehensive manner. Deciphering the molecular basis of flagella-dependent sensorimotricity of unicellular eukaryotes should be rewarding, to understand for example the active and elaborate use of its two flagella to choose their prey by the unicellular chrysophycean alga *E. pulchra* mentioned above [37], the use of cilia cirri by the common fresh water hypotrich *Stylonychia* to walk on a surface ([30], p. 27), or the way in which biflagellates couple the two beatings [50].

Crawling unicellular eukaryotes ensure sensorimotricity through the organization of the actin microfilaments network. This involves a highly connected signalling network between cell surface receptors and most intracellular compartments allowing amoeboid cells to solve complex challenges [110] (see also [88]). Animal cells, like neutrophils chasing a bacterium (https://www.youtube.com/watch?v=I_xh-bkiv_c), are obviously adapting their crawling activity to the movements of their prey. The way in which the actin system is acting on internal polarity cues such as the nucleus-associated centrosome–microtubule network to produce an integrated directional response [99] is not comprehensively understood yet. It is notable that specialized sensory cells in animals have exploited either cilia or actin-dependent structures. This is the case of photoreceptors, for example, with a connecting cilium of the rod or cone cells in vertebrates, and the rhabdomere-containing cells in insects [111].

Relying on the provocative statement by Bray according to which ‘a motile cell is an “intelligent” cell’ ([30], p. 54), one could conclude at least that integrating sensation and motion is basically similar to a reflex action, which happens without the subject thinking. Reflex action has indeed long been proposed to be the first step on the road to a recognizable mind [112]. At the cell level, the issue is to identify the equivalent of the logic elements of the reflex loop from the receptor to the effector.

### 2.4. The cell-organism

Very large (several hundreds of micrometres long) unicellular organisms like ciliates, often with a convex hydrodynamic shape and an anterior–posterior (AP) axis, can swim quite rapidly, having numerous beating cilia covering their body according to a precise and oriented pattern (see [30]). Ciliates belong to the clade of Alveolata in the SAR super-clade, and deserve a specific comment, as their cortical polarity is essentially microtubule-based, with minimal or no contribution from the actin cytoskeleton. They have considerably amplified their flagellar apparatus—thus the number of basal bodies—departing from the usual scheme in unicellular organisms where the nucleus and the flagellar apparatus duplicate in a coordinated manner once per cell cycle. As a consequence, ciliates can demonstrate mechanisms of polarity transmission that are cryptic in other cell types.

Their AP axis represents a unique way to set up cell polarity from rows of polarized elementary units, each formed about a cilium, and a highly complex cortical organization [113] (see also [30]). They were once considered as ‘acellular’ organisms as opposed to uni- or multicellular organisms (A. Lwoff 1978, personal communication). Indeed, similarities between the organization of a *Paramecium* and that of an animal organism have often been noted. Functions devoted to digestive apparatus in animal organisms are ensured by a permanent gullet at the anterior part of the cell, ingesting preys thanks to a potent vortex produced by coordinated beating of numerous cilia, continued by a cytopharynx made of fusing vesicles, and further by a cytoprocte, which ends up in the equivalent of an anus. Two contractile vacuoles function in regulating the water content within the cell, expelling water that contains metabolic wastes, thus ensuring functions devoted to kidneys in animals. Ciliates are also characterized by a nuclear dimorphism, demonstrating a division of labour at the cell level, which is reminiscent of what is observed at the multicellular level. The somatic macronucleus, which develops from the germline micronucleus, is polyploid, containing hundreds of copies of transcribed genes, divides by scissiparity, without mitosis. It controls all cellular functions, including the metabolism [112]. The diploid micronucleus has a germinal function, as it transmits the genome during sexual reproduction. It divides through mitosis at each cell cycle.

**Figure 4.**
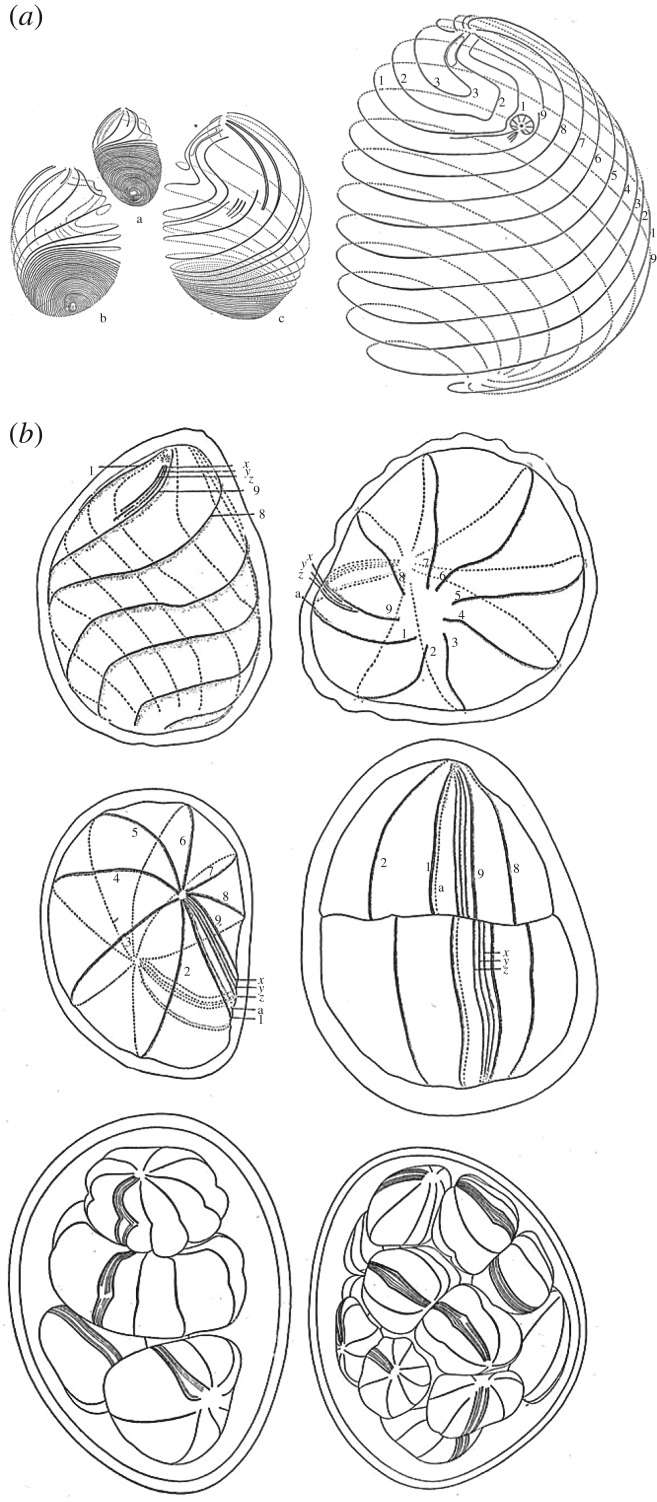
Life cycle of *Foettingeria actiniarum.* Apostomatous ciliates form a very homogeneous group, primarily associated with Crustacea. Along their life cycle, they alternate from spiral to meridian organization of cortical rows of basal bodies when they divide. This is particularly striking in *Foettingeria actiniarum*. (*a*) Growth of the so-called trophont. The detorsion accompanying the increase in size is clealy visible. The smallest cell is 29 µm long, while the next is 60 µm long, and the largest is 160 µm long. (*b*) When detorsion is complete, cells divide several times before forming kysts on the gills if Crustacea (adapted from Lwoff [114]).

Their complex cortex has long made of ciliates appealing models for morphogenesis [114]. The way in which the local cortical polarities, as well as the cell AP axis, are transmitted during the division cycle is quite specific: cells like *Paramecium* divide transversally, in an actin-independent way, at the middle of their AP axis, in such a way that the anterior region assemble a new posterior part, while the posterior part assemble a new anterior part, including a new gullet, so that the two daughter cells keep the AP axis of the mother cell. The old-to-new pole axis corresponds to the ancient AP axis in one daughter cell, while it is the reverse for the other daughter cell. Other ciliates, like the apostomatous ciliates, use basically the same type of division. However, because they grow a great deal during their life cycle, they can show a very complex pattern of divisions depending on species [114] (figure 4). The discovery of ‘cortical inheritance’ or cytotaxis, in *Paramecium* [115], an epigenetic process which confers structural memory of cortical structures for more than 1000 generations (see box 1 in ref. [116]), was the first demonstration that the transmission of polarities involves autonomous mechanisms ensuring structural continuity in addition to mechanisms that rely on genome transmission.

Reproducing a convex body like *Paramecium*, requires the duplication of thousands of basal bodies, which has to be repeated once or twice depending on the region of the cortex, whereas basal bodies in the anterior region of the mother cell, which stays invariant, undertake a specific pattern of abortive duplications [113].

There are countless examples of the critical importance of an AP polarity in ciliates for their survival. A *Didinium* capturing a *Paramecium* (https://www.youtube.com/watch?v=rZ7wv2LhynM) for example, although at sub-millimetric scale in a drop of water, gives the same vivid impression of a predator at metric scale, chasing, biting and eating a prey as big as itself. While the prey *Paramecium* is specifically deciliated at the contact with the predator *Didinium*, the latter uses its own cilia to cope with the movements of the prey trying to escape, and to align the AP axis of the prey with its own AP axis in order to engulf it. The avoidance of an obstacle by *Paramecium* is also an elaborate ciliary-dependent behaviour playing on cell polarity (see [30]). The graded response of a sessile *Stentor* to a noxious food repeatedly applied to its polarized feeding vortex (see [30,110]) demonstrates in addition some sort of cell memory.

### 2.5. The endless plasticity of energy-dependent polarized polymers

Not surprisingly, the dissipative GTP- or ATP-dependent polarized polymers, as well as polymer-dependent molecular motors and other types of polymer-binding proteins, were critical innovations for setting a define polarity in large unicellular eukaryote cells [117,118]. Tubulin and actin proteins are highly conserved in all eukaryotes [118,119]. Tubulins from species as distant as ciliates and vertebrates can co-polymerize *in vivo* [120]. The dynamics of these polymers, due to the molecular properties of their subunits, can be considerably modulated, depending on the cellular context. Actin network plasticity depends on a spectrum of actin-binding proteins in some taxa, and on the repertoire of actin genes in others [119,121]. Actin networks essentially produce forces.

Conspicuous differences that are observed at the cell level, in terms of collective behaviour of microfilaments, are mainly due to a different spectrum of interacting proteins, including those which interact also with cytoplasmic microtubules, when these are present.

Tubulin networks essentially organize cell space by controling spatial distribution of components and of intracellular compartments. Besides the bewildering diversity of binding and motor proteins that have evolved in the different eukaryotic species [122], recent results have significantly expanded the range of intrinsic properties of the microtubules themselves. Microtubule dynamics is usually understood in terms of exchange of *α*/β-tubulin dimers at their ends, fluctuating during their elongation between catastrophe and rescue phases at their (+) ends [123]. They have a very high persistence length and are rectilinear. These properties are apparently exploited in the cortical network of most swimming unicellular eukaryotes, where stable microtubules, also linked all along their length to the plasma membrane, can resist cortex isolation [70]. The doublet microtubules from flagella, bound to the flagellar membrane, are even stiffer, as can be directly observed in splayed cilia when compared to the two individual microtubules of the central pair [124]. Axonemal doublet microtubules, or long-lived microtubules, acquire resistance from mechanical breakage through intraluminal acetylation [125,126]. In animal cells, cytoplasmic microtubules in interphase are often not rectilinear, but curly, crossing each other, forming a very complex network. The recent demonstration of microtubule ability to repair locally lattice defaults in response to mechanical stress opens the way to a new understanding of these polymers properties such as the possibility of a mechano-sensitive assembly of microtubules [127,128].

There is thus a large range of microtubule physical properties that can be modulated in many ways. This could have been critical for cell evolution during the transition from the parietal, membrane-associated pellicle in most unicellular organisms, to the highly versatile organization of centrosome-based intracellular microtubule network in differentiated cells from animal organisms (see §3.3.2.2).

Interestingly, in Bacteria or Archaea cells, there are polarized polymers that are similar to, and thus appear as precursors of, the eukaryotic active polymers [118]. Recently, the discovery of proto-tubulin in Lockiarchae [129], which could bridge the gap between related prokaryotes and eukaryotes—their exact position in the tree of life however is still in debate [130]—has led to interesting speculations on the origin of eukaryotic cell organization [131]. True tubulin has now been discovered in several Archaea, defining a new sub-phylum [132].

## 3. Fate of cell polarity at the transition to multicellularity

The transition from uni- to multicellularity is one of the most puzzling questions in biological evolution, and raises several issues central to Darwinism [133,134]. There is an abundant literature on the possible benefits of being bigger, on selective pressures that favoured this transition in some clades and not in the others, and on the division of labour, corresponding to the number of early cell types in the organism, and taken as a measure of multicellularity and of the coordination it requires [135].

A major question raised by the evolution of multicellularity is how a new unit of selection, demonstrating heritable variations in fitness, is obtained. As stated in [26], conflicts in the founder group of cells have to be resolved, in order to reach a new higher-level unit of selection with increased cooperation among group members and heritable variation in fitness at the group level’. The evolution of multicellular organisms can be indeed opposed by genetic conflicts that arise when mutant cell lineages increase at the expense of the integrity of the multicellular organism. Clonal multicellularity, where the embryo develops from a single cell, and aggregative multicellularity, where there is no feeding and growth during the multicellular stage like in the transient slug of social amoeba in response to starvation, are the two types of possible uni- to multicellular life transitions [85,136]. Clonal multicellularity is the most important defence against genetic conflicts as it minimizes them among cell lineages, and redistributes genetic variation arising within multicellular individuals [137].

Most of the literature on transitions to new units of selection investigates the different forces that favour policing mechanisms for fitness alignment. The possibility that part of this remodelling could rely on cell-based mechanisms has rarely been addressed in the literature of evolutionary theory.

Actually, many unicellular eukaryotes form colonial organizations. This can be observed even in most unexpected cases, such as in Trypanosomes swimming in tight grouping at some challenging stage of their parasitic life cycle [138,139]. The evolutionary benefits of such a strategy might include opportunities for genetic exchange. In all cases, individual cell polarity is a critical parameter of the collective organization, including for bacteria [140].

Going from collectives to permanent multicellular organisms has been successful only in few cases of unicellular eukaryotes [39,141]. Why the rate of success is low, and why multicellularity has led to permanent organisms mainly in two superclades, Archaeplastida (principally seed plants but also independently derived, complex multicellular organisms, like red and brown algae) and Opisthokonta (fungi and animals), has been extensively discussed [84,134,142,143]. Physics of small swimmers shows that their motion can trigger collective features that are different for pushers and pullers: dense populations exhibit a rich collective behaviour at large scales [144]. Whether this has had any role in the differential transitions to multicellularity is not known.

Seed plants and animals are strictly multicellular clades, while fungi are a mix of unicellular (yeasts) and multicellular forms.

### 3.1. Cell-autonomous polarity is not conserved in all multicellular organisms

If we limit the comparison to the three main types of multicellular organisms belonging to Archaeplastids and Opisthokonts, namely seed plants, fungi and animals, they are very different, corresponding actually to quite specific types of multi-cellularity. They also happened at different times during evolution [84]. Indeed, evolving multicellularity from phototrophs and osmotrophs unicellular organisms, where all cells feed like the ancestor, is very different from evolving locomotor multicellular organisms like animals which feed through a centralized mechanism which evolved from the phagocytic feeding mode of the ancestor [39].

#### 3.1.1. Seed plants have lost basal body/axoneme, while volvocales make swimming colonies

Seed plants not only have lost flagella several times, but they have lost all dynein genes as well, like the red algae [145]. Cells have no cell-autonomous polarity [146]. They have a cell sedentary lifestyle, and an actin-dependent intracellular motility. They have a wall made of cellulose, the organization of which depends of intracellular cortical array of microtubules [147]. They do not divide by actin-dependent fission; cell division is incomplete, as cells maintain plasmodesmata between them. Plants tissues are symplasms, in which all cells share the same plasma membrane. There are about 40 early cell types [148].

**Figure 5.**
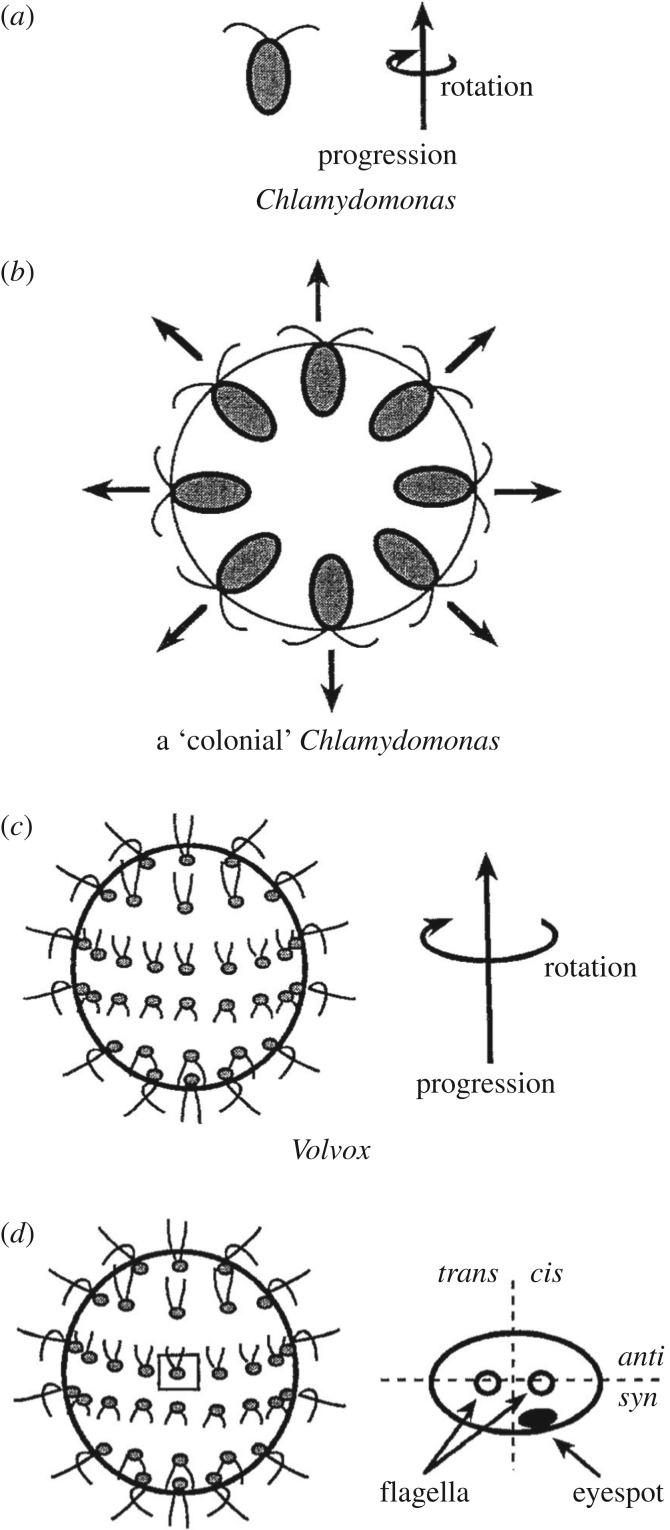
Most motile unicellular organisms display a pattern of motility involving rotation while progressing rectilinearly. This is the case of the single cell *Chlamydomonas* (*a*) and of the colonial multicellular *Volvox* (*c*). If *Chlamydomonas* would form colonies and would keep the same flagellar motion, the colony would not move (*b*). The *Volvox* colony progesses along its antero-posterior axis which corresponds to the antero-posterior axis of individual cells only for those cells positioned in the most anterior part of the colony. All the other cells cannot progress along their own antero-posterior axis, nor can they rotate around their axis. In (*d*) the cellular polarity of each cell (a cell close to the equator of the colony is depicted) will be such that the cellular antero-posterior axis will differ for each position, but the *anti* side will always be towards the colonial anterior pole, thus respecting a planar polarity, as judged by the positions of the eyespots (adapted from Hoops [150]).

Unicellular green algae, like *Chlamydomonas,* are swimming cells with a polar flagellar apparatus with two basal body/axonemes. It is noteworthy that flagella-dependent swimming of unicellular organisms is compatible with the presence of a wall in Archaeplastids, a situation which is not observed in Opisthokonts. Volvocine green algae, like *Volvox carteri*, can form multicellular swimming spherical colonies from unicellular cells like *C. reinhardtii*. They have been extensively used as models of multicellularity, as they display a basic division of labour between peripheral motile somatic cells and internal immotile germ cells [149]. The potential evolutionary benefits of the colonial state are the increased size and the increased swimming speed to exploit spatially distant nutrients or light ressources [150]. The perfectly spherical shape of swimming colonies of individual cells that maintain their association by cytoplasmic bridges, due to incomplete cytokinesis, is not fully understood. It is probably due, however, in one way or another, to the fact that they are colonies of connected swimming cells. Remarkably, each cell demonstrates a shift in the orientation of its nucleus–basal body connector with respect to its body axis, so that each cell can swim cooperatively with the others, the colony swimming as a whole [150,151] (figure 5). This shift reflects the position of each individual cell into the swimming colony, and demonstrates the integration of all cells into a new individual. As a result, the colony has a rotational symmetry, which involves planar polarity as judged by the positioning of the asymmetric eye spot in individual cell [50].

Thus, organismic polarity in swimming colonies of Volvocales is reached by constraining individual cell intrinsic polarities. Policing mechanisms for fitness alignment would involve in that case cell alignment under physical constraints. Whether similar mechanisms of integration exist in metazoans is an open question.

#### 3.1.2. Fungi have evolved a nucleus-associated spindle pole body

Fungi are the product of a quite complex evolution, and comprise eukaryotes with very different life histories, from multicellular species with hyphae, to unicellular organisms like yeasts, the result of secondary transitions [152,153]. ‘Lower’ fungi such as chytrids have flagellated gametes, whereas ‘higher’ fungi have lost basal body/axoneme but have kept dynein genes. The loss of basal body in lower fungi has happened several times and a nucleus-associated spindle pole body (SPB) has evolved instead. In some cases, the SPB retains characters from an ancestral centriole intermediate between ‘lower’ fungi with motile cells and ‘higher’ fungi [154]. In spite of quite a different and simpler structure, the SPB behaves in many respects like the animal centrosome: it nucleates MTs in interphase, duplicates in S phase according to a conservative mechanism [155,156]. Duplicated SPBs are absolutely required for organizing the mitotic spindle, contrary to animal centrosomes, which in some cells, like in oocytes during meiosis, are absent during spindle assembly.

After the loss of the flagellum/basal body and its replacement by the SPB, all stages of fungal life cycle have cell walls [152]. Whether an equivalent correlation is observed in the evolution of metazoans with the apparition of an exoskeleton will be addressed below (in §3.3.2).

Multicellularity in fungi can vary largely, from coenocytic hyphae consisting of continuous cytoplasm with hundreds or thousands of nuclei to septate hyphae with large pores, often with a control of cytoplasm passage between hyphal segments, and also of organelles, including nuclei. Septum formation leading to regularly spaced septa takes place after mitosis in hyphae whereas random septa are observed in other fungi. There is no cell locomotion but intracellular motility, including nuclei migration on great distances, in a dynein-dependent manner, thanks to nucleus-associated SPBs [157]. Cell individuation in hyphae is thus incomplete, with a variable number of nuclei. Interestingly, the gene network regulating dynein activity for nuclear movement in fungi is conserved in vertebrates participating in brain ontogenesis [158].

In unicellular budding or fission yeasts, cell division takes place by actin-dependent fission. Symmetry-breaking of cortical actin microfilaments can be demonstrated in these non-migrating cells, and the way in which the actin network co-aligns with the mitotic spindle, acting as a cell-autonomous polarity cue, transmittable with a generational asymmetry, has been actively studied (see references discussed in [159,160]). Most fungi have a cell wall, made of chitin similar to the chitin from the exoskeleton of arthropods. There are only a few cell types.

#### 3.1.3. Metazoans have evolved a primary cilium/centrosome organ

Metazoans represents only a small fraction of the eukaryotic tree of life (figure 1), yet the diversity of animal forms is considerable. Contrasting with plants or fungi, in which cell shape is maintained by a rigid wall, animal cell shape reflects the spatial organization of internal forces supported principally by the actin cytoskeleton in response to external cues from the extra-cellular matrix (ECM) or from the neighbouring cells [161]. Animal phylogeny today is largely disconnected from the morphological evolution [162]. Metazoans are however unique in their way of making cohesive multicellular tissues by sequential and complete divisions. Animal organisms, with or without an endo- or exoskeleton, are capable of locomotion, their tissues are permanently under forces, and their embryo development in most cases makes an extensive use of directed cell migration during gastrulation and neurogenesis. Cell locomotion—by migration for most somatic cells and by swimming for sperm cells—and morphogenesis of cohesive tissue require cell-autonomous polarity. Through evolution by direct filiation from the ancestral basal body/flagellum, which is otherwise conserved in all species (see [28,29]), a novel and dual organelle appeared, here called ‘primary cilium/centrosome organ’ (PC/C organ), able, depending on the cell state in a given environment, to switch between a plasma membrane-associated primary cilium and a nucleus-associated centrosome. Like in all ophistokonts, cell division takes place by actin-dependent fission. There is no cell wall. There are hundreds of cell types in higher animals, whose evolutionary and functional lineages are far from being clarified [163].

Animal multicellularity has usually been discussed in terms of adhesive innovation [164,165]. Indeed cell–cell contacts and adhesive interaction with the ECM are critical for cell–cell signalling and axis specification during embryogenesis and for building tissues (see §4.2), giving them their plasticity, fluidity and mechanosensitvity [166,167]. However, stable positioning of cells in tissues involve cell autonomous mechanisms, to control cell–cell contacts, adhesive interaction with ECM, orientation of division axis, and the repositioning of daughter cells, including migration of one or both daughter-cells over long distances. There is increasing understanding of the cross-talk between internal and cell adhesive polarity [12–16], as cell–cell contacts and cell adhesion to ECM are accompanied by extensive reorganization of intracellular compartments (see §3.3.2.2).

Actually, our vision of the cell may have to be refined when dealing with cortical versus intracellular polarity. In the early fly syncytial embryo, plasma membrane polarity and compartmentalization have been shown to be established before cellularization [168,169]: despite the absence of plasma membrane boundaries between syncytial nuclei, the secretory membrane system is organized in functionally compartmentalized units around individual nuclei. Thus, whatever the requirement of intact microtubule and F-actin networks, functional equivalents of cells, corresponding to the old concept of ‘energids’ (see [170]), were demonstrated with modern tools, in the complete absence of plasma membrane boundaries within a syncytium. Earlier work on the same system demonstrated that centrosomes alone were able to divide and reorganize microtubules, actin and spectrin networks, as well as plasma membrane of polar cells in the syncytium [171]. Centrosomes could therefore play a major role in organizing cytoplasm. Other examples are known [172] (see §3.3.2.1).

The possibility that the dual PC/C organ is a critical innovation for preserving cell-autonomous polarity during the transition to multicellularity in Metazoa is thus worth exploring.

### 3.2. Is the conservation of cell-autonomous polarity at the origin of Metazoa?

Of the five super-clades, Opisthokonta and Amoebozoa are sister groups forming the former unikonts, now called Amorphea. Interestingly, centrosomes, or centrosome-like organelles, have evolved mainly in Amorphea [41]. In close relatives of animals and fungi, evolutionary convergence of lifestyles (as judged by loss or apparition of swimming ability, apparition of a filopodiated form, or of osmotrophy with a cell wall) was achieved apparently through differential retention of genomic characters from the common ancestor of fungi and animals [173].

Several scenarios have been proposed for the transition to metazoans. Besides the old Gastrae hypothesis [142], the so-called synzoospore hypothesis due to Zakhvatkin [174] was recently revisited and promoted in the light of novel genomic data defining new groups of unicellular eukaryotes related to Metazoa [84]. It was noted very early (in the nineteenth century) that sponge choanocytes were very similar to choanoflagellates, having also the same feeding mode, thus suggesting that sponges evolved from a choanoflagellate. This view has been apparently quite supported by phylogenetic methods (see [39]). The rare ability of choanoflagellate cells to stick together in a colony, yet to still feed as before, is taken as a strong argument for making choanoflagellates animal ancestors: sponges could thus have evolved directly from unicellular organisms without changing feeding mode. The issue of whether Poriphora or Ctenophora is sister to the other Metazoa is however much debated and proving hard to resolve.

Selective pressures proposed to have favoured uni- to multicellular transition are of two general types. First, the benefit of large size that would make it easier to escape from predation; as a matter of fact, adding a predator in a culture of individual choanoflagellates rapidly triggers colony formation [175]. Second, the benefits of cooperation, either in the so-called flagellar synthesis constraint according which cells that stop moving when dividing would have by-passed this constraint through interaction with moving non-dividing cells like in Volvox [26,176], or in the formation of filtration/feeding structures [177,178].

When did the PC/C organ first appear? Interestingly, the presence of non-motile (9 + 0) primary cilia that use calcium channels has been clearly established in the osculum of sponges, a chimney-like structure through which water exits [179]. Experimental evidence suggests that the osculum functions as a sensory system to detect changes in flow and control whole sponge responses. Thus, Poriphora lack conventional muscles and nerves, yet sense and respond to changes in their fluid environment. Such an organized array of primary cilia could represent the first step in the evolution of sensory and coordination systems, suggesting that selective pressure for sensation was at the origin of multicellular organisms ability of their individual cells to trigger responses to the environment in a coordinated manner. This could be also an early example of planar cell polarity in animals (see §4.2).

Whatever the scenario, a reasonable working hypothesis could be that selective pressure to maintain cell-autonomous polarity in Metazoa—necessary for individual cells to sense the environment and trigger responses in a coordinated manner, or to be positioned in a concerted manner in tissues—would have favoured the transition from the basal body/flagellum of swimming unicellular organisms to a new organ in amoeboid cells, able to adopt two interconvertible versions in individual cells, depending on their environment or on their proliferation state: the plasma membrane-associated non beating primary cilium, and the nucleus-associated basal body/centriole-based centrosome organelle.

This transition would have been progressive in the animal multicellular lineage. Indeed, many swimming ‘lower’ animals, like cnidarians, have most of their cells with a beating cilium (see §4.2).

The new PC/C organ, which maintains either one of the two end connections of the ancestral nucleus basal body connector, would have been selected on the same sensory-motricity integrated function than the ancestral basal body/flagellum, but in different cells, or in the same cell at different moments. It would also transmit intrinsic cell polarity during cell division [172]. In addition to cooptation/innovation of specific genes, new connections in signalling pathways, adapted to the new cytoskeletal organization, would have taken place. Apparently, any sort of transition from flagellate to amoeboid cell organization was possible, as shown by examples in extant organisms (see §2.2). The animal amoeboid cell organization had to ensure a permanent cross-talk between the new organ and a wall-less actin-based cell cortex in individual migrating cells, as well as in cells building specialized contacts with neighbour cells in tissues. In both cases, these cells have to be exquisite mechanosensors [180,181], able to shape ‘soft’, mechanosensitive tissues in organisms capable of locomotion.

In most ‘higher’ animal species, the beating activity of the ancestral flagellum was kept only in the male germ cell line, or in specialized multiciliated epithelial cells. In both cases, the transition from centrosome to basal bodies, or vice versa at fertilization, involves specific processes [182]. But, indeed, mono-ciliated or mono-flagellated cells are very common in ‘lower’ animals. They are a majority in cnidarians (see §4.2).

### 3.3. The primary cilium/centrosome organ allows a context-dependent switch in cell polarity architecture

Many and detailed reviews have been recently published on the different functions of the primary cilium [104,183–194] and of the centrosome organelle [172,195–214], including several collective coverages on each organelle. I will address only few points that are important for this perspective.

#### 3.3.1. The primary cilium, a single-copy sensory organelle of critical importance in many post-mitotic cells

The views on the role of the primary cilium in cell economy went up and down since its first description in the nineteenth century [215]. It was proposed by Henneguy and by Lenhossek, at the end of the nineteenth century, that the pair of dots, the so-called centrioles, within the centrosome, as seen by light microscopy after silver staining, could be the same organelles as the dots at the base of the cilia/flagella, the so-called kinetosomes at the time. The hypothesis was rapidly accepted on growing indirect evidence, but had to wait until electron microscopic observation to be definitively validated. In addition the two dots appeared, unexpectedly, as two small cylinder-shaped structures with a ninefold radial symmetry. Remarkably, it is the genetic and biochemical analysis of flagellar growth in the unicellular green algae *C. reinardtii* that has led to the current accumulation of original results on human ciliopathies [183], showing clearly that ‘the time of primary cilium has come’ [215]. Quoting a recent review ([194], pp. 126, 138): ‘Cilia mediate an astonishing diversity of processes … the logic of this combinatorial signalling represents one of the most important challenges to the ciliogenesis field … the cellular “antenna”, far from being a passive receiver of input signals, is due for an upgrade to the status of a cellular “central processing unit”, and perhaps the main one integrating extracellular signalling with the cell cycle and metabolism.’ Is there any specific role of the basal body itself for such a quite important function? A similar function—as hub for signalling integration and cell cycle control—has been proposed for the centrosome [206]. Analysis of the evolution of the interactions at the centriole–basal body transition should bring key informations [216,217].

#### 3.3.2. The centrosome, a single-copy organelle, in search of a comprehensive definition

A general definition of the centrosome in different models has been difficult until now. As stated by K. Gull at the EMBO Centrosome meeting 2017 in Heidelberg, paraphrasing, with due apologies, Winston Churchill on his famous comment on Russia:^2^ ‘It is difficult to understand the actions of the centrosome. It is a riddle (the centriole) wrapped in a mystery (the PCM) inside an enigma (the organization of the cytoplasm and cytoskeleton): but perhaps there is a key.’ If it exists, the key has to be looked for in comparing either different species during evolution, or differentiating cells during development in the same species. The key must cope with the fact that, besides common properties such as a conservative duplication once per cell cycle that creates a generational asymmetry, centrosome actions are context-dependent. As emphasized elsewhere ([218], p. 7), ‘one cannot hope to get at a comprehensive understanding of centrosome function in diverse systems without a comparative analysis of the cellular economy resulting from the survival strategy of each organism’.

The general consensus has long been that the primary function of centrioles in animal cells was to template cilia or flagella and that their role, if any, in the centrosomes at the mitotic spindle poles, was a secondary one (for an analysis of this view point, see [219,220]). However, with an evolutionary scenario in which the PC/C organ of multicellular organisms has evolved by direct filiation from the flagellum of unicellular ancestors, one is tempted to think it is the other way around: it is because basal bodies in unicellular organisms not only template axonemes but also control the whole cell division process from their parietal position (see §2.1.4) that these two functions could be embodied in two different versions of the ancestral organelle in the multicellular context. One version, the primary cilium, is maintaining a parietal position when cells are quiescent and in contact with the interstitial medium, while the other one, the centrosome, maintains a juxtanuclear position when cells are growing, or acting as circulating cells by polarized signalling and communication with other cells, like blood cells, or are located within a compact tissue. The animal transition from uni- to multicellular organism would have imposed two types of basal body-based organelles in order to accommodate topological constraints in the multicellular organism, for tissue growth and for cell sensorimotricity.

#### 3.3.2.1. Comparing species

The evolutionary history of centrosomes has progressed considerably in recent years [40,221–223], but is probably far from being fully comprehensive, as we do not have good data from enough species. We lack a unifying description of the functions of the centrosome, which is suggested nevertheless by the remarkable structural conservation of the centriole-basal body throughout the evolution of eukaryotes. The centrosome, seen by Van Beneden as the dynamic centre of the cell, was seen by Boveri as the division organ, *coordinating* karyokinesis and cytokinesis. Most later studies, however, were limited to deciding whether the centrosome had any role in segregating chromosomes, when it appeared that chromosomes could be segregated in its absence, as in seed plants, or in female meiosis for most animal organisms. That the centrosome, when present, improves the fidelity of chromosomes segregation has been now experimentally supported [224]. It is now known that acentriolar mice die at mid-gestation, 24 h before those that cannot make cilia, indicating that centrioles have other functions than acting only as a basal body [225].

*Centrosome and cell cycle progression*. Karyokinesis and cytokinesis are the outcome of a long cell cycle progression. There is increasing evidence that centrosome has a critical role in the temporal control of the major transitions of the cell cycle progression (see [226,227]). The recent demonstration that an attenuated mitotic clock is controlling the assembly of basal bodies in post mitotic multiciliated cells without any effect on the nuclear compartment [228] suggests a specific and sensitive regulation of centrioles duplication by the CDK1/APC oscillator that could be important in setting the correct temporal order of events during the cell cycle progression. In fission yeast, the centrosome/SPB has been shown to integrate inputs from multiple pathways to control cell decisions at G2–M–G1 transitions [229]. The possibility to block centrosome duplication by inhibiting PlK4 activity has revealed a p53-dependent sensing of this block, or of the prolonged mitosis that is triggered by this block, and an arrest in G1 (see references in [230]). Transformed cells, in which p53 is inactivated, keep growing, although at reduced rate. The analysis of these cells by lens-free microscopy, which allows robust statistics on thousands of cells and cell lineage analysis, has revealed a strong asymmetry for daughter cells in both cell cycle duration and cell size, suggesting a link with the generational asymmetry of the centrosome (C. Allier and M. Bornens 2017, unpublished data). The behaviour of the mother and the daughter centriole all along the cell cycle progression was shown previously to be quite different [231,232].

In the intracellular apicomplexae parasites, the cell cycle demonstrates a surprising flexibility: cells can produce a progeny from two to thousands of cells. In *Toxoplasma*, it is the parasite centrosome which segregates the functions of karyokinesis and cytokinesis, thanks to a bipartite organization with two asynchronously replicating core complexes with distinct localization, composition and function [233]. In very large eggs from many marine and amphibian organisms, cell division represents an extraordinary challenge in terms of spatial and temporal organization. Simple molecular diffusion is incompatible with the very rapid division rate [234]. It has been argued that spatial and temporal organizational challenges may be solved by chemical reaction waves: the centrosome, rather than simply nucleating microtubules by structural templating, would play a key role in organizing these waves by initiating two autocatalytic reactions that travel across the large cytoplasm as chemical waves. Waves of microtubule-stimulated microtubule nucleation would propagate out from centrosomes while using the Cdk1 oscillator to coordinate the timing of cell division. In this view, the centrosome would rapidly organize the large cytoplasm during the short embryonic cell cycle. An argument for this view is that *X. laevis* egg activation (by pricking or any other means) triggers the sequential 13 biochemical oscillations of Cdk1 activity, but neither egg organization nor cleavage occurs. Cleavage and parthenogenetic development is observed only if a centrosome is injected when activating the egg [235].

Other experimental demonstration of the morphogenetic role of the centrosome in eggs exist, such as the centrosome-driven cleavages of enucleated blastomeres in the early development of starfish eggs after enucleation [172] (figure 2), or rapid divisions of membrane-less cell bodies in the fly syncytial embryo [168]. In this system, centrosomes alone are able to divide and reorganize microtubules and actin networks as well as plasma membrane of polar cells, after aphidicolin treatment, or in a mutation in the *gnu* locus in which centrosomes and nuclei are dissociated [171]. This morphogenetic role suggests that the centrosome acts as a signalling centre, a role that is supported by results in somatic cells [236]. Recent results suggest that such a role might expand, as the centrosome has been recently shown to nucleate actin [237,238], or to recycle endosomes [239]. The ability of the centrosome-based aster to physically integrate the cell as a unit at cytokinesis, by locally bending the plasma membrane around itself, through its microtubule organizing activity, has been video-recorded directly in the amoeba *D. discoideum* [240]. When supernumerary nucleus-associated bodies (NABs), a simpler type of nucleus-associated centrosome which nucleates and permanently anchors a low and constant number of microtubules, are elicited, they duplicate and behave like fully competent centrosomes, except that they are not associated with the nucleus. At cytokinesis, when the two daughter cells form around the two nucleus-associated NABs, each supernumerary nucleus-free NAB organizes a cytoplast around itself by the same mechanism, and in this way is eliminated.

The main metaphors used to describe the functions of the centrosome, such as cell division organ or cell dynamic centre in the early days, and more recently cell individuation organ, signalling centre or stress sensor, to name a few, emphasize different actions of the centrosome. Experimental lines of evidence supporting one or another metaphor are however largely overlapping. Altogether, the difficulty of getting at a simple description of the centrosome actions is due to its involvement in both temporal and spatial aspects of cell activity, through its generational asymmetry, and the participation of microtubules in controlling the cellular distribution of cell compartments and active complexes, and possibly in cell-size sensing, or in cell sizing [241,242].

*The puzzling case of Ecdysozoa.* As far as centrosomes are concerned, the fly *Drosophila melanogaster* and the worm nematode *C. elegans* represent a paradox*.* The genetic analysis of early development in these two models, which may not be representative of the most common development of Ecdysozoa, has yet been of invaluable importance for identifying the conserved core complex of gene products necessary for centriole duplication [203], as well as key regulatory activities controlling the centrosome maturation and activity at G2–M–G1 transition [207,209,213]. Yet, somatic centrioles—the situation is apparently different for basal bodies from the sperm cells in flies [243]—are not canonical in these two species, and look as simplified versions of the highly conserved centriolar structure, lacking the distal part where appendages are anchored on the mother centriole. As a matter of fact, they lack from one-third in the fly to half in the worm of gene products that are conserved in both unicellular organisms and mammalians [223]. They lack for example homologues of the rare genes that are conserved between the human centrosome and the yeast SPB, such as cdc31 or Sfi1, which are, nevertheless, critical for the yeast SPB duplication and present in vertebrates and unicellular organisms [28,52,244,245]. In cultured cells from *Drosophila*, centrosomes apparently recruit γ-tubulin only in G2/M, and contrary to centrosomes from different animal species, those from *Drosophila* are unable to trigger parthenogenetic cleavage in *Xenopus* eggs [246]. Moreover, although centrosomes are essential during the early syncytial stage [247], apparently normal development takes place in the absence of functional centrosomes [248] or of centrosomes at all [249]. As flies, in that case, are not viable, because they are unable to grow cilia in sensory neurons, this surprising result was taken as an argument for proposing that centrioles are dispensable for somatic cell division and required only for acting as basal bodies. Further work has, however, demonstrated a more canonical view, revealing that in acentrosomal flies, centrosomes play vital roles in spindle assembly, function and orientation in epithelial tissues [250]. This work has however demonstrated in flies the existence of multiple mechanisms buffering the effects of centrosome loss. More generally, hexapodes are known for the atypical size, structure and motility of their flagella spermatozoa [251]. And the only examples in which the constraints on the ninefold symmetry are relaxed to produce highly divergent and enlarged microtubule-based centrioles are in the male germ line of some insect groups [252]. An intriguing correlation has been noted in this work between those divergent centrosomes and the unusual reproductive system in these insects, in which all chromosomes of paternal origin are eliminated from the male germ line.

Thus, the duplication machinery identified in flies and nematodes is highly conserved in all other species, as expected, but other putative common centrosome- or centriole-associated functions that could be present in other metazoans, and in most unicellular organisms, are missing. Could this puzzling situation allow us to anticipate the kind of common functions that would be dispensable at the centrosome in flies and nematodes? If one would have to take a stand, the first thing coming to mind is that Ecdysozoa, contrary to the other metazoans, have an exoskeleton, whose rigidity and stiffness is probably accommodating most of the forces exerted on the tissues. The cuticle is tighly associated with the apical pole of cells through huge proteins under tension [253]. Would this alleviate forces that are exerted on cells in tissues of other species, and *in fine* on the centrosome in cells, through cytoskeletal structures attached to the distal part of centrioles? Centrioles have been shown to resist MT-dependent forces exerted on centrosomes during mitosis [254]. Do they also support forces during interphase when they are proliferating, or during quiescence when they are in tissues? Interestingly, this possibility could shed light on the mechanisms triggering the decentration of the centrosome observed in differentiated cells in many tissues from vertebrate organisms.

#### 3.3.2.2. Comparing cells during development of the same species

In individual cells, the centrosomal microtubule aster often coexists with microtubules nucleated at the Golgi apparatus [255,256]. Cell differentiation is generally associated with microtubule reorganization into non-centrosomal microtubule arrays [257,258]. The centrosome is no longer at the cell centre. Microtubules, which are normally nucleated and anchored at the centrosome [259], are either nucleated at the centrosome and further transported for anchoring elsewhere, or directly nucleated elsewhere [260]. This reorganization can involve a specific relocalization of some centrosomal proteins at cell–cell junctions which is mandatory for epithelial cell polarization [261]. In most cases, the new microtubule network seems well adapted to support the differentiated cell shape. But how to explain that the centriole pair, which often does not nucleate microtubules as most PCM has been shed, is no longer at the cell centre, but rather in specific positions, for example, close to the cell periphery?

If the centralized position of the centrosome in isolated cells reflects the balance of forces exerted on it [262], then, as soon as two cells adhere tightly to each other, they form a new object on which forces need to be balanced, forcing the centrosome in one or in both cells to move towards the other cell. This happens for example when killer cells adhere to a target cell [263], or when cell confinement, which controls the extent of ECM adhesion affordable to cells, triggers tight cell–cell contacts, and modulates centrosome position and the polarity of a pair of epithelial sister cells [14]. Conversely, the two centrosomes located towards the centre of a pair of epithelial cells interacting with each other go back to the centre of each cell when they separate from each other, before they undertake epithelial-to-mesenchymal transition [16]. In this view, one would expect the position of the centrosome in each cell of a growing tissue during embryo development to change progressively as cells differentiate. The fact that, in many differentiated cells, the centriole pair no longer nucleates or anchors microtubules would suggest that forces are no longer directly exerted on the centrosome through microtubules. Actin microfilaments would be a good candidate to participate in this function, as the centrosome can become a very efficient actin microfilaments nucleator when microtubule nucleation is toned down [237]. A competition between plasma membrane-associated adhesive structures and the centrosome for actin nucleating factors such as Arp2/3 has been demonstrated in lymphoid cells [238], as well as the requirement of the Rho-associated protein kinase p160ROCK for centrosome positioning [264]. A role of actin cytoskeleton in anchoring basal bodies at the apical plasma membrane during ciliogenesis in multiciliated epithelia is well documented [265,266]. In differentiating muscle cells, where centrosomes from myoblasts are eliminated [267], large forces exerted on tissues during muscle activity would be accommodated at the tissue level.

## 4. Cell autonomous polarity and the evolution of individuality

**Figure 6.**
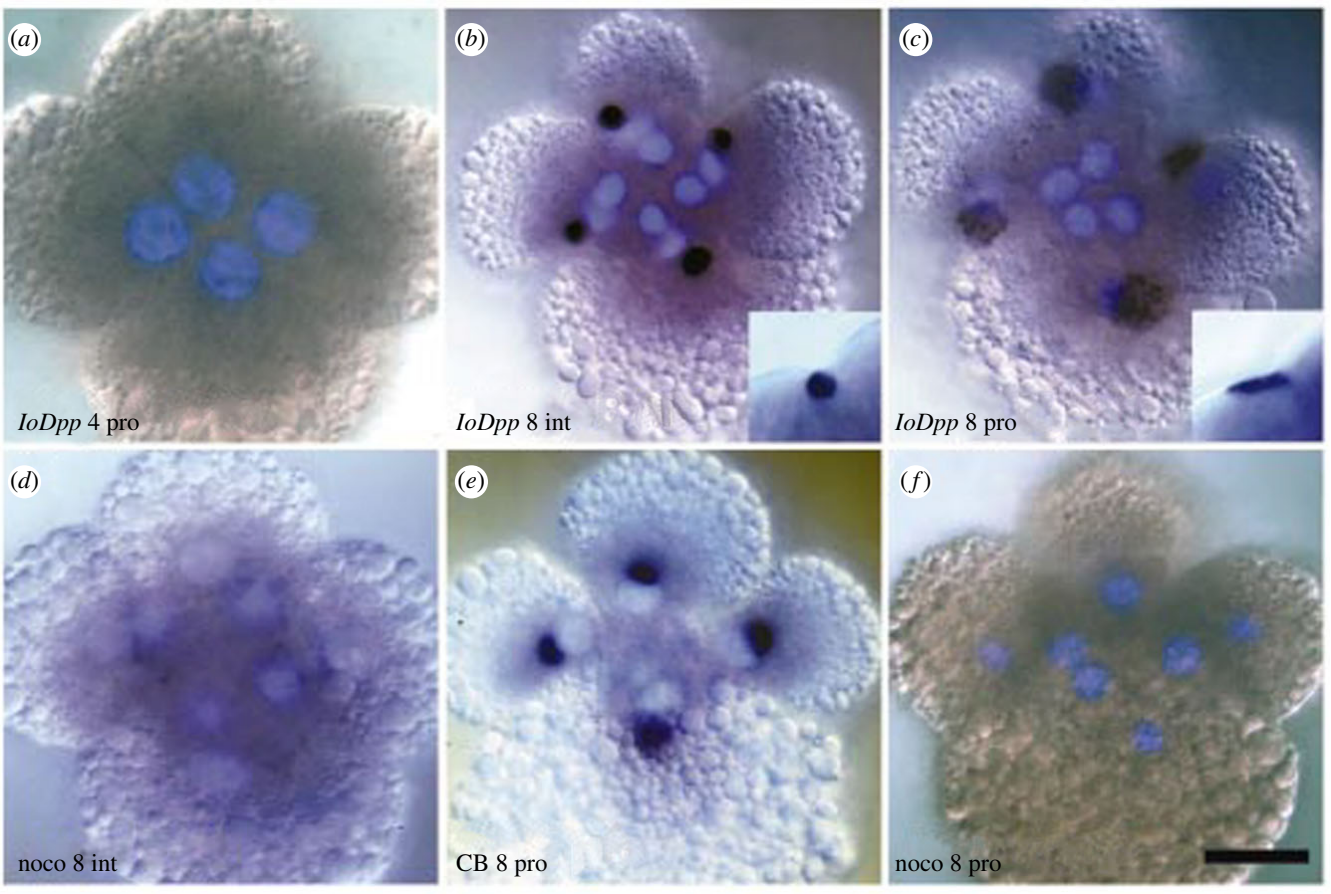
Asymmetric inheritance of centrosomally localized mRNAs during embryonic cleavages in the mollusc *Ilyanassa obsoleta* (adapted from Lambert & Nagy [268]).

Besides re-establishing diploidy, egg fertilization in many species involves the resetting of egg polarity through the conversion of the sperm head-associated basal body into the embryo centrosome (see [172]). Whatever the correct functional definition of centrosomes that would accommodate all the data accumulated on evolutionary distant experimental models, all centrosomes display a generational asymmetry that is heavily exploited during development to distribute cell fate determinants, like mRNAs during embryonic spiral cleavages [268] (figure 6), or to maintain stem cell pools [6,7]. More recently, centrosomal asymmetry has been shown to control the Notch pathway in neural progenitors by specifically recruiting a regulator of the Notch pathway asymmetrically [269]. Remarkably, this work also demonstrated a compensatory mechanism restoring symmetry in proliferative divisions, which involves a pool of the same regulator associated with the Golgi apparatus, the other microtubule-nucleating organelle in many cells.

A critical event for the fate of the species during development is the transmission of the germ line. Does the generational asymmetry of the centrosome have any role in this transmission?

### 4.1. How is the germ line transmitted during development?

The way in which germ cells are specified is highly variable in different species [270,271]. However, early specification of the germ line is a critical contention of the Weismann's doctrine of ‘the continuity of the germ plasm’, at the basis of the modern synthetic theory of evolution [26]. Remarkably, early terminal differentiation is far from being a general feature [134]. It is a character limited to some higher metazoan taxa: ‘Individuality is a derived character’ [26].

**Figure 7.**
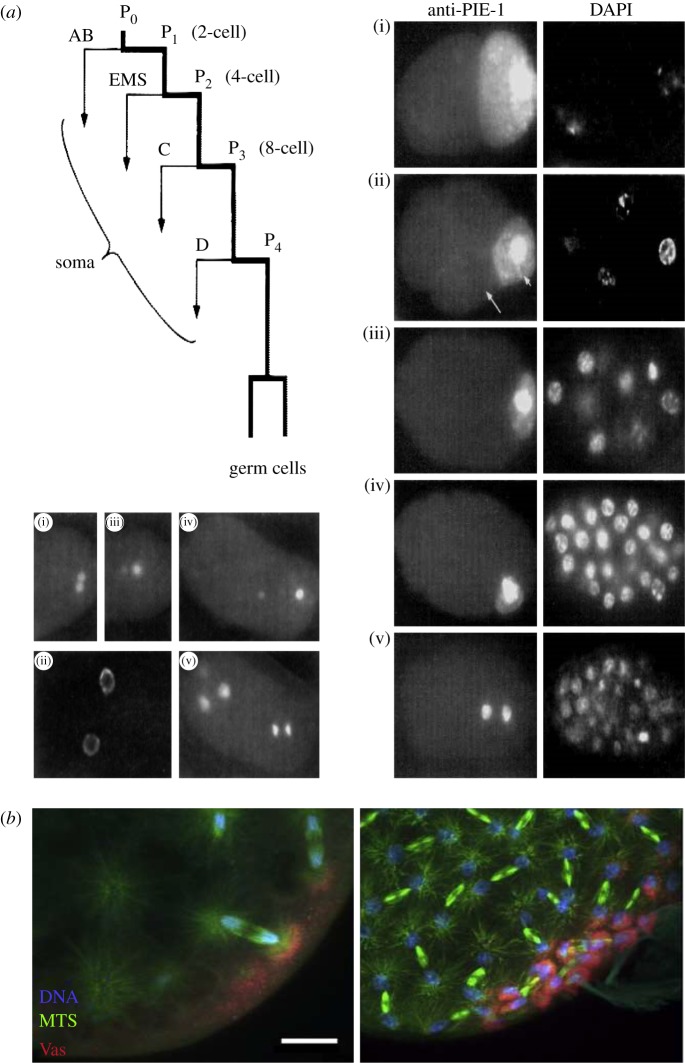
(*a*) The PIE-1 protein and germline specification in *C. elegans* embryos. PIE-1 protein is concentrated on centrosomes at both spindle poles and is further degradated asymmetrically after rotation by 90° of the posterior mitotic spindle (top row on the bottom left frame). After mitosis, PIE-1 maintains totipotency in the posterior blastomere (right frame) by repressing the somatic state (adapted from Mello *et al*. [272]). (*b*) Asymmetrical transport of germ plasm (Vasa in red) on astral microtubules (green) towards one spindle pole (left) directs germ cell development in *Drosophila* (right) (adapted from Lerit & Gavis [273]).

In nematodes and flies, where germ plasm transmission has been documented, germ cells form very early, during early egg development, according to very distinct mechanisms. Both mechanisms, however, depend critically on the orientation of the mitotic spindle and of the generational asymmetry between the two poles [272,273] (figure 7). Treating fly syncitial embryos with aphidicolin had previously demonstrated that centrosomes alone were sufficient to initiate the formation of pole cells [274]. In most animals, once specified, the primordial germ cells migrate into the developing gonads where they will undertake meiosis and gametogenesis. In early oocytes of most animals, including humans, Balbiani bodies are conspicuous asymmetric non-membrane bound compartments accumulating mitochondria, reticulum, Golgi membranes and mRNA for use in the egg after fertilization [275]. They disperse once oocytes are activated. How such composite accumulations can be maintained has long been a mystery. They have been shown recently to be stabilized by a so-called physiological amyloid (i.e. a reversible form of amyloid that could be involved in preserving dormancy in vertebrate oocytes) [276]. In the zebrafish oocyte, Balbiani body formation and oocyte polarization are apparently coupled to meiotic chromosomal pairing during the conserved bouquet stage by the oocyte centrosome [277–279]. The bouquet stage is when all chromosome telomeres are concentrated and attached to the nuclear periphery facing the centrosome. In most animal species, when the oocyte is activated, its centrosome is eliminated during meiosis [280]. The embryo polarity is reset by the sperm cell: indeed, male meiosis does not eliminate the centrosome but uses it extensively for sperm cell differentiation, transforming centrioles into basal bodies to template the flagellum.

Although far from comprehensively described in most models, preserving cell polarity during male and female gametogenesis is apparently critical.

The coupling between oocyte polarization and the chromosomal bouquet by the centrosome during oogenesis, as observed in the zebrafish, and possibly in other models [281], suggests the possibility of a more permanent coupling between intra-nuclear organization, and the activity of the sensory-motor apparatus as a polar organ, acting as the essential mediator between the cell and its environment. This domain is explored mainly in the primary cilium which is specialized for Hedgehog signal transduction [184–194]. Promising hints exist also in bacteria [282]. This sensory transduction adds to the rapidly progressing field of mechano-transduction in multicellular organisms [283].

### 4.2. Regeneration and asexual reproduction: how are they often associated?

The ability of animals to regenerate missing parts is a fascinating property, quite variable among species and still poorly understood. There is however a considerable literature on regeneration ability. Regeneration usually goes together with asexual reproduction (see for example [284–286]): ‘Generally, those animals that undergo asexual reproduction are also the ones that are able to regenerate extremely well, both anteriorly and posteriorly … Species capable of regenerating anteriorly are also able to regenerate posteriorly. The opposite is not true; there is a number of species that can regenerate posteriorly, but not anteriorly’ ([287], p. 260). This description, which mainly concern worms of the protostomian Lophotrochozoa branch indicates that regeneration ability respects the body polarity. ‘In obligatory sexually reproducing animals the regeneration capacity is generally considerably diminished, with plenty of examples from flatworms, annelids and nemerteans’ ([287], p. 260). Is there any cellular feature that could shed some light on the association of asexual reproduction with the ability to regenerate the body in respecting its polarity? Is the evolution of the PC/C organ having any role in the common association of regeneration and asexual reproduction?

Flatworms like planarians are indeed known, since early experiments in the eighteenth century, for their remarkable capacity to regenerate, thanks to pluripotent stem cells at the origin of all cell types [284]. One single neoblast can rescue a lethally irradiated animal. An asexual strain of *Schmidtea mediterranea* is able to reproduce asexually forever: the body split in two parts, each end regenerates the missing part [288, 289]. The genome of *S. mediterranea*, which was long resistant to classical sequencing, has been very recently assembled, thanks to several new technical approaches [290]. A wealth of important informations on genome organization has been gained for a better understanding of the regeneration capacity of these animals. In addition, and remarkably, planarians have lost a number of essential genes, among which are MAD1 and MAD2, the core components of the spindle assembly check-point (SAC), as well as numerous other SAC components. MAD1 and MAD2 are highly conserved, including in other flatworms. Apparently, planarians have evolved a SAC-like response in the absence of these core components [290]. This remarkable absence of the classical SAC is probably related to another remarkable loss in the evolution of planarians, namely that of the PC/C organ [291] (see also [172]). As the dynamics of the SAC are highly dependent on the localization of the different complexes, the loss of centrosomes at the spindle poles in neoblasts had probably an impact on the whole network.

Thus, although planarians glide on beating cilia from multiciliated epithelial cells, and produce biflagellated sperm cells, they lack centrosomes or primary cilia in other cells, including in the dividing neoblasts. All basal body/centriole genes found in unicellular organisms or in metazoans, including those that are missing in Ecdysozoa, are present, as expected from the presence of multiciliated epithelial cells. However, a few genes encoding key components for centrosome formation, such as SPD2/Cep192, are missing [291]. Spiral cleavage, which characterizes this clade, is lost in planarians. All these features are specific of planarians: the flatworm *Macrostomum lignano* has centrosomes in the dividing neoblats, reproduces sexually, develops by spiral cleavage and regenerates poorly. It would be important indeed to know if planarians features can be observed in other highly regenerative animals.

Can one interpret these data in the framework of the evolution of multicellularity? Is it possible that metazoan multicellular organisms display, in a more cryptic way, the equivalent of the policing strategy observed in Volvox colonies, where the organismic polarity imposes changes to the polarity axis of individual cells according to their position in the colony (see §3.1.1 and figure 5)? In other words, is it possible that part of the remodelling necessary for organogenesis is triggered by early conflicts among individual cell-autonomous polarities that need to be resolved to preserve a global polarity of the organism?

**Figure 8.**
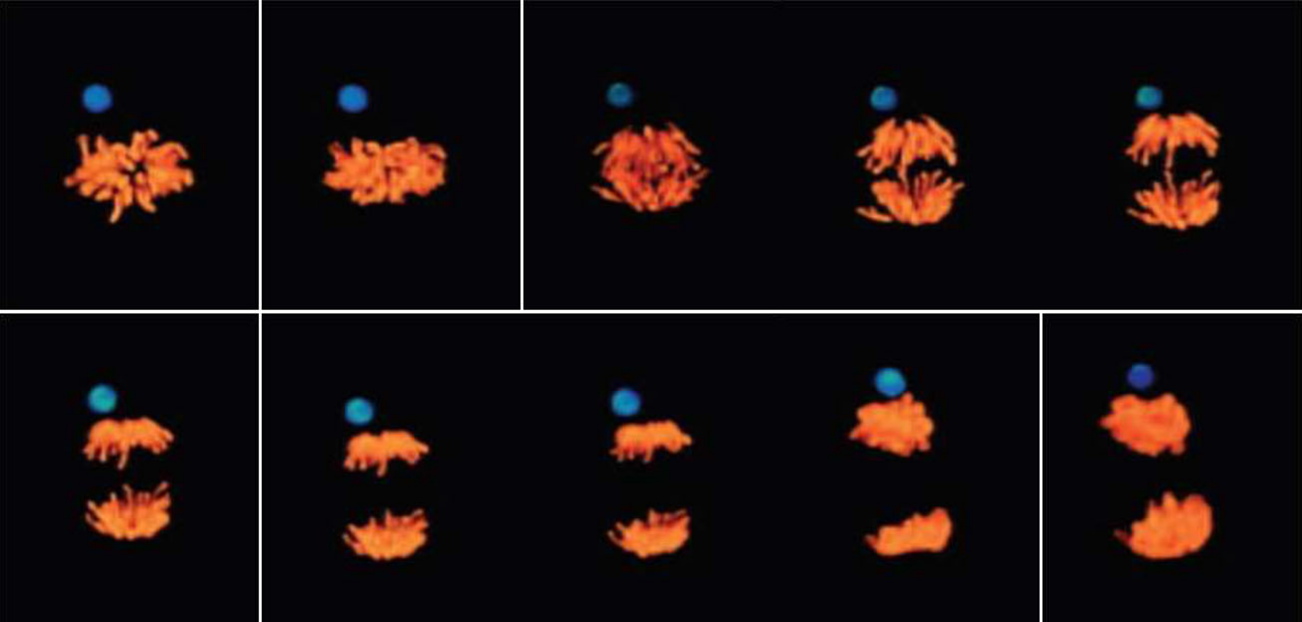
A localized Wnt signal orients asymmetric stem cell division *in vitro*. Images from three-dimensional time-lapse microscopy of segregating chromosomes in ES cells expressing H2B-Venus that were cocultured with Wnt3a beads (blue) (adapted from Habib *et al*. [294]).

Most animals use canonical Wnt signalling to control their A–P body axis. The axial patterning role for Wnt signalling is very ancient. It pre-dates the evolution of bilaterally symmetric animals, as it also influences primary axis polarity of cnidarians [292]. Posterior Wnt signalling and anterior Wnt inhibition are conserved in protostome and deuterostome axial patterning, including in planarians. The axis of polarization in planarians is independent of tissue movements and developmental events that occur during gastrulation in other species. Planarians re-establish axial identities in regeneration, by high β-catenin activity at posterior-facing wounds and low β-catenin activity at anterior-facing wounds. The fact that planarians use this pathway in A–P axis regeneration could indicate a role of Wnt signalling in regulating positional features [292]. As a matter of fact, in *C. elegans*, where development does not occur via patterning of tissue fields like in most species, but via highly reproducible cell lineages, Wnt signalling controls the polarity of individual asymmetric cell divisions. In arthropods, it establishes segment polarity [292]. On the other hand, β-catenin has been shown to localize to centrosomes, where it would promote mitotic progression [293]. It binds to, and is phosphorylated by, Nek2, an important kinase controlling centrosome cohesion. β-Catenin interacting proteins involved in Wnt signalling, such as adenomatous polyposis coli, Axin and GSK3β, are also localized at centrosomes where they would also promote mitotic progression. It has also been noted that proteins that regulate mitotic spindle positioning, such as dynein, are associated with cell–cell adhesion sites and cell cortex, thus suggesting a possible role of β-catenin from there [293]. Finally, to mimic developmental signals that are often presented to cells in an oriented manner, Wnt-coated beads were used to localize signalling at the single-cell level [294]. This induced asymmetric distribution of Wnt-β-catenin signalling components, oriented the plane of mitotic division and directed asymmetric inheritance of centrosomes (figure 8).

Planar coordination of individual cell polarity with neighbouring cells is also necessary. Planar cell polarity (PCP) is a key feature of many adult tissues, involving the Wnt membrane receptor frizzled (Fz), and acting as a compass defining and coordinating polarity in static and moving cells [295]. PCP accounts for the common orientation of structures such as hairs or feathers. It is crucial for controlling basal body orientation and for coordinating ciliary beating direction in many vertebrate multiciliated epithelia [295]. Work on cnidarians established that the Fz–PCP pathway is probably also an ancestral metazoan feature that arose in association with multicellularity. Evolutionary arguments suggest that Fz–PCP is responsible for oriented swimming and feeding in relation to body axis in the many ciliated larval types in the animal lineage [295,296]. This raises the interesting possibility that the Fz–PCP pathway and Wnt/β-catenin-dependent axis specification could have been coordinated in early metazoans by the participation of a common Wnt ligand: Fz not only mediates cytoplasmic/nuclear signalling responses to extracellular Wnt via control of cytoplasmic β-catenin degradation, but also allows coordination of planar polarity between adjacent cells [295]. Strikingly, certain core PCP components were apparently not present in the unicellular organisms from which animals evolved. Their appearance during the evolution of multicellularity in the animal lineage suggests a role in coordinating more ancient cell polarity characters within tissues. Wnt appeared with animal multicellularity, whereas components involved in cell shape and motility were present in ancestral unicellular organisms [295]. Many links have been shown between cellular processes like ciliogenesis, apical docking of basal bodies or cell intercalation during gastrulation and the Wnt/Fz signalling.

As a whole, PCP development along the principal body axis in the early embryo might have had important consequences for body plan evolution by allowing coordination along the body axis of cell behaviours during morphogenetic processes, such as gastrulation and elongation of the embryo [295].

Thus, centrioles or basal bodies, as critical intrinsic cues of cell-autonomous polarity, could be used by developmental signals as tools to position cells during development. In the absence of these cell-autonomous polarity cues, like in the body of planarians, the axial patterning by Wnt signalling coordinated to the Fz–PCP signalling would go unperturbed, even after physical interruption by sectioning. Fz–PCP would keep controlling apical basal body anchoring and cilia formation in the polarized basal multiciliated epithelium [292]. This would preserve the cell generation potential, allowing the body to regenerate from its parts through adult pluripotent cells.

In other animals, the axial patterning by Wnt signalling would be modified all along, whenever cells, with their intrinsic polarity, would respond to the signalling, and modify their positioning or the orientation of their divisions, their cell cycle duration or gene expression. This would lead to coherent organs, built on cells positioned in such a way that all individual cell polarities would be correctly oriented at the organ level. Each tissue has its own stem cells. They act as lineage-progenitor cells, exploiting the asymmetry of the PC/C organ in a specific way, for growth or for controlled migration. This would represent a step-wise solution, elaborated during embryo development, allowing individual cell-autonomous polarities and organismic polarity to be compatible. However, the correlate would be that the organism could not regenerate from its parts, nor reproduce asexually.

In this view, the differential ability of organisms for tissue regeneration would have to be found at the cell level, in the ability of cells to express a more or less strong cell-autonomous polarity, able to respond to axial patterning.

Indeed, the precise involvement of core PCP proteins in controlling morphogenesis might have changed during animal evolution depending on the varying contribution of different cellular processes to the morphogenetic events of embryogenesis [295]. For example, cnidarians frequently reproduce both asexually and sexually, have high regenerative capacity and have organs with tissues and compopent cells well organized through PCP with coordonated ciliated beating (E Houliston 2017, personal communication).

### 4.3. Sensorimotricity integration, brain development and mate choice

A remarkable result of recent years was that most known MCPH genes responsible for primary microcephaly in humans encode a protein having some role in the primary cilium or in the centrosome, suggesting specific PC/C organ-dependent constraints during brain development [205]. Indeed, neurons are exceedingly polarized, and the brain is certainly the organ in which cell polarity is exploited to its ultimate possibilities. Before briefly analysing how the development of the vertebrate nervous system could help understanding brain susceptibility to mutations in PC/C organ proteins, let us look at brain development in an evolutionary perspective, as the development of a courtship machine for optimal reproduction.

Sexual selection by mate choice is an important part of natural selection, for higher animals. We know that Darwin struggled for most of his life to interpret mate choice strategies, such as the famous example of the peacock tail: it is a pretty and efficient courtship machine to seduce hens, when the peacock erects it in a trembling row to diffract light, but a heavy and dangerous load otherwise. Darwin, who had first to accept that females could have the power to choose their mates, concluded that choosing based on the beauty of the male tail meant that the sense of beauty was not unique to human beings, but had an evolutionary basis, which could be traced to birds [297]. Since that time, many aspects of Darwin's proposals on sexual choice have been revisited by modern research, and his ideas on the existence of a human-like sense of aesthetic in animals are no longer accepted as such, the evolution of female choice being identified now as a key topic for study [298]. Maynard Smith, who studied courtship in flies, where females evaluate the strength of males on their ability to perform the dance they impose, proposed that females would most often directly detect components of fitness [299]. Thus, in this view, erecting such a tail for a peacock is hard work and hens would estimate the strength of the males in this way. In any case, the survival advantage in investing in a pretty tail is clearly limited compared with investing in a big brain as a courtship machine. In addition, the success of the mating process is critically dependent on sensorimotricity coordination. Cephalization corresponds to the concentration of sensory structures at the anterior end of the body in bilaterians. As for unicellular organisms, the anterior end of a moving animal is normally the first to encounter food, danger or other stimuli. The human species has evolved a distinctly big brain.

During the development of the vertebrate central nervous system, neurogenesis is a very complex process, in which the spatial and temporal balance between proliferation and differentiation is precisely regulated. Mitotic spindle orientation plays a key role in this regulation [300,301]. A link between centrosome asymmetry, primary ciliogenesis and daughter cell fate has been demonstrated [80]. Daughter cells, which maintain stem cell identity in the mouse neocortex, preferentially inherit the mother centriole, which mediates the nucleation of the primary cilium. The mother centriole is able to retain ciliary membrane, thus allowing an early cilium reformation which results in earlier ciliary signalling in the corresponding cell. Moreover, reformation of primary cilia in nascent differentiating daughter cells takes place at the baso-lateral side instead of the apical side, and this is apparently the first identifiable cell state before neural progenitor delamination from the apical adherens junction belt of the neuroepithelium [301]. Delamination of epithelial cells is critical for tissue morphogenesis. Apparently, from this work on mouse neuroepithelium, a re-orientation of cell polarity, by changing the mother-centriole docking site, precedes delamination. This provides a direct example of the role of PC/C organ in cell polarity reorientation during morphogenesis. Indeed, temporal and spatial asymmetries in ciliogenesis could lead to differential exposure of cells to signalling from the environment, and thus to the amplification of differential fates.

The development of an integrating sensorimotor organ like the brain, involves, therefore, many specific PC/C organ-dependent constraints, either on centrosome-dependent division process, or on primary cilium-dependent signalling reception, or in both, that could explain brain particular susceptibility to mutations in genes governing the assembly or the activity of the PC/C organ.

Interestingly, it has been proposed that the control of cilia-dependent locomotion could be at the origin of neural circuits [302]. At the pluteus larval stage of many marine animals, like starfish, evolutionarily highly conserved linear arrays of beating cilia move the multicellular larvae while attracting planktonic prey [303]. As for unicellular organisms (see §2), a trade-off between feeding and swimming can be observed, which varies depending on the environment, and which potentially implicates neuronal control of cilia. It could be the actual selective pressure for high evolutionary conservation of these complex larval linear arrays of beating cilia.

## 5. Conclusion

This survey started with the contention that sensorimotricity integration is the main evolutionary constrained function imposing a polarized organization of unicellular ancestors, and that the basal body/flagellum had a key role in transmiting this function at each generation, allowing in addition an efficient feeding of the moving cell. This survey ends up discussing the role of the PC/C organ, evolved by direct filiation from the ancestral basal body/flagellum in Metazoa, in the morphogenesis of the organ responsible for the centralized integration of sensorimotricity in humans. It is an example of the evolutionary conservation of a constrained functional module, leading to selected survival solutions similar at the uni- and the multicellular scale, with the same molecular or cellular mechanisms. This survey has also been discussing the potential role of the cellular PC/C organ in the establishment of biological individuality in metazoans. The same PC/C organ turns out to have a critical role in brain development, and thus in the potential development of a behavioural individuality.

Thus, beyond critical consequences for embryo development and tissue morphogenesis, the conservation of cell-autonomous polarity at the multicellular transition and its transmission through divisions, based on the innovation of the centrosome/primary cilium switch and on the conservative mode of basal body-centriole reproduction, could have had far reaching implications for the evolution of individuality in higher metazoans.

At the end of this attempt to look for a comprehensive understanding of the significance of a cell-autonomous symmetry breaking in animal cells, two questions appear in more urgent need of answers. The first one concerns the evolutionary origin of the eukaryotic basal body–flagellum. In the absence of any traces of previous attempts before the highly conserved structure in all extant eukaryotes, there are only interesting speculations so far on an endogenous scenario [38,43,44,223,304], as opposed to the unlikely proposal of a symbiotic origin [176]. Knowing more on the origin of this derived character of eukaryotes would certainly illuminate many aspects of its function in cell polarity. Knowing, for example, whether it appeared early during eukaryogenesis, independently or not of the endosymbiosis which led to mitochondria that were essential for the prokaryote–eukaryote transition [305], would be of critical importance. Was there a coupling between the two innovations in kinetoplastidae like Trypanosoma, belonging to the ancient Excavata supergroup, which have their mitochondrion tightly associated with the basal body, the reproduction of which governs the segregation of the kinetoplast DNA [58]?

The second question concerns the activities of the centriole-basal body organelle. At the end of a recent and thorough analysis of the literature, emphasizing the variations that can occur in different species (or even in the same species) in the otherwise highly conserved centriole organelle, two experts concluded that until a better understanding of ‘the nuances of the molecular pathways that operate in physiological cellular contexts’, and ‘because of the diversity in their number, structure, and function … centrioles will rightfully remain a central enigma in cell biology’ ([306], p. 830). I hope this Perspective will help give a framework for a better understanding of the centriole/basal body organelle in cells and organisms during evolution.

Perhaps a further crack in the enigma could come from raising and finding the way to address experimentally questions that are rarely asked. For example, what sort of global intra-centriole–basal-body dynamics are required to shape a centrosome with its highly dynamic PCM, and to recruit a whole series of associated structures and protein complexes in the right spatial and temporal order during the cell division cycle? Does the basal body not only structurally template the primary cilium, or the flagellum, but also participate in the control of the considerable intraciliary signalling and dynamics within an extremely confined environment, or in the control of the flagellar beating? And if so, how?

## Endnotes

1 The term ‘individuality’ is used here in its usual sense, as defined by LW Buss in *The evolution of individuality* [26].

2 ‘I cannot forecast to you the action of Russia. It is a riddle wrapped in a mystery inside an enigma: but perhaps there is a key. That key is Russian national interest’ (Winston Churchill, radio broadcast, 1 October 1939).

## Data accessibility

This article has no additional data.

## References

[1] Alexandre C, Baena-Lopez A, Vincent J-P 2014 Patterning and growth control by membrane-tethered Wingless. Nature 505, 180–185. (10.1038/nature12879) 24390349 PMC7611559

[2] Etoc F *et al.* 2016 A balance between secreted inhibitors and edge sensing controls gastruloid self-organization. Dev. Cell. 39, 302–315. (10.1016/j.devcel.2016.09.016) 27746044 PMC5113147

[3] Yamashita YM, Mahowald AP, Perlin JR, Fuller MT 2007 Asymmetric inheritance of mother versus daughter centrosome in stem cell division. Science 315, 518–521. (10.1126/science.1134910) 17255513 PMC2563045

[4] Gonzalez C 2007 Spindle orientation, asymmetric division and tumour suppression in *Drosophila* stem cells. Nat. Rev. Genet. 8, 462–472. (10.1038/nrg2103) 17510666

[5] Januschke J, Gonzalez C 2010 The interphase microtubule aster is a determinant of asymmetric division orientation in *Drosophila* neuroblasts. J. Cell Biol. 188, 693–706. (10.1083/jcb.200905024) 20194641 PMC2835941

[6] Pelletier L, Yamashita YM 2012 Centrosome asymmetry and inheritance during animal development. Curr. Opin Cell Biol. 24, 541–546. (10.1016/j.ceb.2012.05.005) 22683192 PMC3425708

[7] Reina J, Gonzalez C 2014 When fate follows age: unequal centrosomes in asymmetric cell division. Phil. Trans. R. Soc. B 369, 20130466 (10.1098/rstb.2013.0466) 25047620 PMC4113110

[8] Drubin DG, Nelson WJ 1996 Origins of cell polarity. Cell 84, 335–344. (10.1016/S0092-8674(00)81278-7) 8608587

[9] Johnston DS, Ahringer J 2010 Cell polarity in eggs and epithelia: parallels and diversity. Cell 141, 757–774. (10.1016/j.cell.2010.05.011) 20510924

[10] Nance J 2014 Getting to know your neighbor: cell polarization in early embryos. J. Cell Biol. 206, 823–832. (10.1083/jcb.201407064) 25267293 PMC4178963

[11] Román-Fernández A, Bryant DM 2016 Complex polarity: building multicellular tissues through apical membrane traffic. Traffic 17, 1244–1261. (10.1111/tra.12417) 27281121

[12] Gupta SK, Meiri KF, Mahfooz K, Bharti U, Mani S 2010 Coordination between extrinsic extracellular matrix cues and intrinsic responses to orient the centrosome in polarizing cerebellar granule neurons. J. Neurosci. 30, 2755–2766. (10.1523/JNEUROSCI.4218-09.2010) 20164359 PMC2846173

[13] Feldman JL, Priess JR 2012 A role for the centrosome and PAR-3 in the hand-off of MTOC function during epithelial polarization. Curr. Biol. 22, 575–582. (10.1016/j.cub.2012.02.044) 22425160 PMC3409831

[14] Rodríguez-Fraticelli AE, Auzan M, Alonso MA, Bornens M, Martín-Belmonte F 2012 Cell confinement controls centrosome positioning and lumen initiation during epithelial morphogenesis. J. Cell Biol. 198, 1011–1023. (10.1083/jcb.201203075) 22965908 PMC3444774

[15] Jiang T, McKinley RFA, McGill MA, Angers S, Harris TJC 2015 A Par-1-Par-3-centrosome cell polarity pathway and its tuning for isotropic cell adhesion. Curr. Biol. 25, 2701–2708. (10.1016/j.cub.2015.08.063) 26455305

[16] Burute M et al. 2017 Polarity reversal by centrosome repositioning primes cell scattering during epithelial-to-mesenchymal transition. Dev. Cell 40, 168–184. (10.1016/j.devcel.2016.12.004) 28041907 PMC5497078

[17] Théry M, Racine V, Piel M, Chen Y, Sibarita J-B, Bornens M 2005 The extracellular matrix guides the orientation of the cell division axis. Nat. Cell Biol. 7, 947–953. (10.1038/ncb1307) 16179950

[18] Théry M, Bornens M 2006 Cell shape and cell division. Curr. Opin Cell Biol. 18, 648–657. (10.1016/j.ceb.2006.10.001) 17046223

[19] Fink J et al. 2011 External forces control mitotic spindle positioning. Nat. Cell Biol. 13, 771–778. (10.1038/ncb2269) 21666685

[20] Morin X, Bellaïche Y 2011 Mitotic spindle orientation in asymmetric and symmetric cell divisions during animal development. Dev. Cell 21, 102–119. (10.1016/j.devcel.2011.06.012) 21763612

[21] Ragkousi K, Gibson MC 2014 Cell division and the maintenance of epithelial order. J. Cell Biol. 207, 181–188. (10.1083/jcb.201408044) 25349258 PMC4210436

[22] Bellaïche Y 2016 Cell division in the light of modeling. Dev. Cell 38, 584–586. (10.1016/j.devcel.2016.09.008) 27676430

[23] Gonzalez C 2013 *Drosophila melanogaster*: a model and a tool to investigate malignancy and identify new therapeutics. Nat. Rev. Cancer 13, 172–183. (10.1038/nrc3461) 23388617

[24] Harold FM 2014 In search of cell history: the evolution of life's building blocks. Chicago, IL: University of Chicago Press.

[25] Anderson PW 1972 More is different. Science 177, 393–396. (10.1126/science.177.4047.393) 17796623

[26] Buss LW 1987 The evolution of individuality. Princeton, NJ: Princeton University Press.

[27] Adl SM et al. 2012 The revised classification of eukaryotes. J. Eukaryot. Microbiol. 59, 429–493. (10.1111/j.1550-7408.2012.00644.x) 23020233 PMC3483872

[28] Azimzadeh J, Bornens M 2004 The centrosome in evolution. In Centrosomes in development and disease (ed. Nigg E), pp. 93–121. New York, NY: Wiley.

[29] Bornens M, Azimzadeh J 2007 Origin and evolution of the centrosome. Adv. Exp. Med. Biol. 607, 119–129. (10.1007/978-0-387-74021-8_10) 17977464

[30] Bray D 2001 Cell movements: from molecules to motility, 2nd edn Boca Raton, FL: CRC Press.

[31] Bowman GR, Lyuksyutova AI, Shapiro L 2011 Bacterial polarity. Curr. Opin Cell Biol. 23, 71–77. (10.1016/j.ceb.2010.10.013) 21095111 PMC7500059

[32] Jarrell KF, McBride MJ 2008 The surprisingly diverse ways that prokaryotes move. Nat. Rev. Microbiol. 6, 466–476. (10.1038/nrmicro1900) 18461074

[33] Ginger ML, Portman N, McKean PG 2008 Swimming with protists: perception, motility and flagellum assembly. Nat. Rev. Microbiol. 6, 838–850. (10.1038/nrmicro2009) 18923411

[34] Cavalier-Smith T 2014 The neomuran revolution and phagotrophic origin of eukaryotes and cilia in the light of intracellular coevolution and a revised tree of life. Cold. Spring Harb. Perspect. Biol. 6, a016006 (10.1101/cshperspect.a016006) 25183828 PMC4142966

[35] Benmerah A 2013 The ciliary pocket. Curr. Opin Cell Biol. 25, 78–84. (10.1016/j.ceb.2012.10.011) 23153502

[36] Clerot J, Iftodea F, Budina K, Jeanmaire-Wolfa R, Coffeb G, Fleury-Aubussona A 2001 Fine oral filaments in *Paramecium*: a biochemical and immunological analysis. J. Eukaryot. Microbiol. 48, 234–245. (10.1111/j.1550-7408.2001.tb00310.x) 12095113

[37] Wetherbee R, Andersen AR 1992 Flagella of a chrysophycean alga play an active role in prey capture and selection. Protoplasma 166, 1–6. (10.1007/BF01320137)

[38] Saraste J, Goud B 2007 Functional symmetry of endomembranes. Mol. Biol. Cell. 18, 1430–1436. (10.1091/mbc.e06-10-0933) 17267686 PMC1839002

[39] Cavalier-Smith T 2017 Origin of animal multicellularity: precursors, causes, consequences-the choanoflagellate/sponge transition, neurogenesis and the Cambrian explosion. Phil. Trans. R. Soc. B 372, 20150476 10.1098/rstb.2015.0476) 27994119 PMC5182410

[40] Yubuki N, Leander BS 2013 Evolution of microtubule organizing centers across the tree of eukaryotes. Plant J. 75, 230–244. (10.1111/tpj.12145) 23398214

[41] Azimzadeh J 2014 Exploring the evolutionary history of centrosomes. Phil. Trans. R. Soc. B 369, 20130453 10.1098/rstb.2013.0453) 25047607 PMC4113097

[42] Saito A et al. 2003 Gliding movement in *Peranema trichophorum* is powered by flagellar surface motility. Cell Motil. Cytoskeleton 55, 244–253. (10.1002/cm.10127) 12845598

[43] Sung C-H, Leroux MR 2013 The roles of evolutionarily conserved functional modules in cilia-related trafficking. Nat. Cell Biol. 15, 1387–1397. (10.1038/ncb2888) 24296415 PMC4016715

[44] Mitchell DR, Cilia E 2017 Evolution of Cilia. Cold. Spring. Harb. Perspect. Biol. 9, a028290 10.1101/cshperspect.a028290) 27663773 PMC5204320

[45] Lecointre G, Le Guyader H 2001 *Classification phylogénétique du vivant*. Paris, France: Editions Belin.

[46] Balestra FR, von Tobel L, Gönczy P 2015 Paternally contributed centrioles exhibit exceptional persistence in *C. elegans* embryos. Cell Res. 25, 642–644. (10.1038/cr.2015.49) 25906994 PMC4423087

[47] Fritz-Laylin LK, Levy YY, Levitan E, Chen S, Cande WZ, Lai EY, Fulton C 2016 Rapid centriole assembly in Naegleria reveals conserved roles for both de novo and mentored assembly. Cytoskeleton 73, 109–116. (10.1002/cm.21284) 26873879

[48] Beech PL, Heimann K, Melkonian M 1991 Development of the flagellar apparatus during the cell cycle in unicellular algae. Protoplasma 164, 23–37. (10.1007/BF01320812)

[49] Moestrup HT 1989 Ultrastructure of the flagellar apparatus in *Pyramimonas octopus* (Prasinophyceae). II. Flagellar roots, connecting fibres, and numbering of individual flagella in green algae. Protoplasma 148, 41–56. (10.1007/BF01403990)

[50] Wan KY, Goldstein RE 2016 Coordinated beating of algal flagella is mediated by basal coupling. Proc. Natl Acad. Sci. USA 113, E2784–E2793. (10.1073/pnas.1518527113) 27140605 PMC4878519

[51] Nohynková E, Tumová P, Kulda J 2006 Cell division of *Giardia intestinalis*: flagellar developmental cycle involves transformation and exchange of flagella between mastigonts of a diplomonad cell. Eukaryot. Cell 5, 753–761. (10.1128/EC.5.4.753-761.2006) 16607022 PMC1459668

[52] Salisbury JL, Baron A, Surek B, Melkonian M 1984 Striated flagellar roots: isolation and partial characterization of a calcium-modulated contractile organelle. J. Cell Biol. 99, 962–970. (10.1083/jcb.99.3.962) 6381510 PMC2113404

[53] Lechtreck KF, Melkonian M 1991 Striated microtubule-associated fibers: identification of assemblin, a novel 34-kD protein that forms paracrystals of 2-nm filaments in vitro. J. Cell Biol. 115, 705–716. (10.1083/jcb.115.3.705) 1918160 PMC2289178

[54] Brugerolle G, Mignot J-P 2003 The rhizoplast of chrysomonads, a basal body-nucleus connector that polarises the dividing spindle. Protoplasma 222, 13–21. (10.1007/s00709-003-0016-4) 14513307

[55] Gely C, Wright M 1985 Centriole size modifications during the cell cycle of the amoebae of the mxyomycete *Physarum polycephalum*. J. Ultrastruct. Res. 91, 127–137. (10.1016/0889-1605(85)90064-3) 2868129

[56] Gräf R, Batsios P, Meyer I 2015 Evolution of centrosomes and the nuclear lamina: amoebozoan assets. Eur. J. Cell Biol. 94, 249–256. (10.1016/j.ejcb.2015.04.004) 25952183

[57] Bornens M 1977 Is the centriole bound to the nuclear membrane? Nature 270, 80–82. (10.1038/270080a0) 927525

[58] Ogbadoyi EO, Robinson DR, Gull K 2003 A high-order trans-membrane structural linkage is responsible for mitochondrial genome positioning and segregation by flagellar basal bodies in trypanosomes. Mol. Biol. Cell 14, 1769–1779. (10.1091/mbc.e02-08-0525) 12802053 PMC165075

[59] Gluenz E, Povelones ML, Englund PT, Gull K 2011 The kinetoplast duplication cycle in *Trypanosoma brucei* is orchestrated by cytoskeleton-mediated cell morphogenesis. Mol. Cell. Biol. 31, 1012–1021. (10.1128/MCB.01176-10) 21173163 PMC3067821

[60] Paintrand M, Moudjou M, Delacroix H, Bornens M 1992 Centrosome organization and centriole architecture: their sensitivity to divalent cations. J. Struct. Biol. 108, 107–128. (10.1016/1047-8477(92)90011-X) 1486002

[61] Beisson J, Wright M 2003 Basal body/centriole assembly and continuity. Curr. Opin Cell Biol. 15, 96–104. (10.1016/S0955-0674(02)00017-0) 12517710

[62] O'Toole ET, Giddings TH, McIntosh JR, Dutcher SK 2003 Three-dimensional organization of basal bodies from wild-type and delta-tubulin deletion strains of *Chlamydomonas reinhardtii*. Mol. Biol. Cell 14, 2999–3012. (10.1091/mbc.e02-11-0755) 12857881 PMC165693

[63] Dupuis-Williams P et al. 2002 Functional role of epsilon-tubulin in the assembly of the centriolar microtubule scaffold. J. Cell Biol. 158, 1183–1193. (10.1083/jcb.200205028) 12356863 PMC2173240

[64] Wang JT, Kong D, Hoerner CR, Loncarek J, Stearns T 2017 Centriole triplet microtubules are required for stable centriole formation and inheritance in human cells. Elife 6, e29061 (10.7554/eLife.29061) 28906251 PMC5653238

[65] Azimzadeh J, Hergert P, Euteneuer U, Formstecher E, Khodjakov A, Bornens M 2009 hPOC5 is a centrin-binding protein required for assembly of full-length centrioles. J. Cell Biol. 185, 101–114. (10.1083/jcb.200808082) 19349582 PMC2700515

[66] Pazour GJ, Agrin N, Leszyk J, Witman GB 2005 Proteomic analysis of a eukaryotic cilium. J. Cell Biol. 170, 103–113. (10.1083/jcb.200504008) 15998802 PMC2171396

[67] Amos LA, Löwe J 2017 Overview of the diverse roles of bacterial and archaeal cytoskeletons. Subcell. Biochem. 84, 1–26. (10.1007/978-3-319-53047-5_1) 28500521

[68] Jarrell KF, Albers S-V 2012 The archaellum: an old motility structure with a new name. Trends Microbiol. 20, 307–312. (10.1016/j.tim.2012.04.007) 22613456

[69] Farr H, Gull K 2012 Cytokinesis in trypanosomes. Cytoskeleton 69, 931–941. (10.1002/cm.21074) 23027706

[70] Sherwin T, Gull K 1989 Visualization of detyrosination along single microtubules reveals novel mechanisms of assembly during cytoskeletal duplication in trypanosomes. Cell 57, 211–221. (10.1016/0092-8674(89)90959-8) 2649249

[71] Sunter JD, Varga V, Dean S, Gull K 2015 A dynamic coordination of flagellum and cytoplasmic cytoskeleton assembly specifies cell morphogenesis in trypanosomes. J. Cell Sci. 128, 1580–1594. (10.1242/jcs.166447) 25736289 PMC4406125

[72] Perasso L, Brett SJ, Wetherbee R 1993 Pole reversal and the development of cell asymmetry during division in cryptomonad flagellates. Protoplasma 174, 19–24. (10.1007/BF01404038)

[73] Holmes JA, Dutcher SK 1989 Cellular asymmetry in *Chlamydomonas reinhardtii*. J. Cell Sci. 94, 273–285. 2621224 10.1242/jcs.94.2.273

[74] Feldman JL, Geimer S, Marshall WF 2007 The mother centriole plays an instructive role in defining cell geometry. PLoS Biol. 5, e149 (10.1371/journal.pbio.0050149) 17518519 PMC1872036

[75] Karpov SA 2016 Flagellar apparatus structure of choanoflagellates. Cilia 5, 11 (10.1186/s13630-016-0033-5) 27148446 PMC4855756

[76] Théry M, Bornens M 2008 Get round and stiff for mitosis. HFSP J. 2, 65–71. (10.2976/1.2895661) 19404473 PMC2645575

[77] Shapiro L 1993 Protein localization and asymmetry in the bacterial cell. Cell 73, 841–855. (10.1016/0092-8674(93)90266-S) 8500178

[78] Raikov IB 1994 The diversity of forms of mitosis in protozoa: a comparative review. Eur. J. Protozol. 30, 253–269. (10.1016/S0932-4739(11)80072-6)

[79] Anderson CT, Stearns T 2009 Centriole age underlies asynchronous primary cilium growth in mammalian cells. Curr. Biol. 19, 1498–1502. (10.1016/j.cub.2009.07.034) 19682908 PMC3312602

[80] Paridaen ML, Wilsch-Bräuninger M, Huttner WB 2013 Asymmetric inheritance of centrosome-associated primary cilium membrane directs ciliogenesis after cell division. Cell 155, 333–344. (10.1016/j.cell.2013.08.060) 24120134

[81] Taniguchi K et al. 2017 An apicosome initiates self-organizing morphogenesis of human pluripotent stem cells. J. Cell Biol. 216, 3981–3990. (10.1083/jcb.201704085) 29021220 PMC5716285

[82] Fuentealba LC, Eivers E, Geissert D, Taelman V, De Robertis EM 2008 Asymmetric mitosis: Unequal segregation of proteins destined for degradation. Proc. Natl Acad. Sci. USA 105, 7732–7737. (10.1073/pnas.0803027105) 18511557 PMC2402384

[83] Rujano MA et al. 2006 Polarised asymmetric inheritance of accumulated protein damage in higher eukaryotes. PLoS Biol. 4, e417 (10.1371/journal.pbio.0040417) 17147470 PMC1750924

[84] Sebé-Pedrós A, Degnan BM, Ruiz-Trillo I 2017 The origin of Metazoa: a unicellular perspective. Nat. Rev. Genet. 18, 498–512. (10.1038/nrg.2017.21) 28479598

[85] Brunet T, King N 2017 The origin of animal multicellularity and cell differentiation. Dev. Cell 43, 124–140. (10.1016/j.devcel.2017.09.016) 29065305 PMC6089241

[86] Brunet T, King N 2017 International workshop, Nov 7–9, 2017: Origins of Metazoa, Aviesan, Paris.

[87] Fritz-Laylin LK, Lord SJ, Mullins RD 2017 WASP and SCAR are evolutionarily conserved in actin-filled pseudopod-based motility. J. Cell Biol. 216, 1673–1688. (10.1083/jcb.201701074) 28473602 PMC5461030

[88] Bonner JT 2010 Brainless behavior: a myxomycete chooses a balanced diet. Proc. Natl Acad. Sci. USA 107, 5267–5268. (10.1073/pnas.1000861107) 20332217 PMC2851763

[89] Fritz-Laylin LK et al. 2010 The genome of *Naegleria gruberi* illuminates early eukaryotic versatility. Cell 140, 631–642. (10.1016/j.cell.2010.01.032) 20211133

[90] Walsh CJ 2007 The role of actin, actomyosin and microtubules in defining cell shape during the differentiation of *Naegleria* amebae into flagellates. Eur. J. Cell Biol. 86, 85–98. (10.1016/j.ejcb.2006.10.003) 17189659

[91] Smith HE 2014 Nematode sperm motility. WormBook 1–15. (10.1895/wormbook.1.68.2) PMC5402208 24715710

[92] Bernheim-Groswasser A, Wiesner S, Golsteyn RM, Carlier M-F, Sykes C 2002 The dynamics of actin-based motility depend on surface parameters. Nature 417, 308–311. (10.1038/417308a) 12015607

[93] Bernheim-Groswasser A, Prost J, Sykes C 2005 Mechanism of actin-based motility: a dynamic state diagram. Biophys. J. 89, 1411–1419. (10.1529/biophysj.104.055822) 15923234 PMC1366625

[94] Verkhovsky AB, Svitkina TM, Borisy GG 1999 Self-polarization and directional motility of cytoplasm. Curr. Biol. 9, 11–20. (10.1016/S0960-9822(99)80042-6) 9889119

[95] Jékely G 2014 Origin and evolution of the self-organizing cytoskeleton in the network of eukaryotic organelles. Cold. Spring. Harb. Perspect. Biol. 6, a016030 (10.1101/cshperspect.a016030) 25183829 PMC4142967

[96] Wickstead B, Gull K 2011 The evolution of the cytoskeleton. J. Cell Biol. 194, 513–525. (10.1083/jcb.201102065) 21859859 PMC3160578

[97] Rogers SS, Waigh TA, Lu JR 2008 Intracellular microrheology of motile *Amoeba proteus*. Biophys. J. 94, 3313–3322. (10.1529/biophysj.107.123851) 18192370 PMC2275677

[98] Callan-Jones AC, Voituriez R 2016 Actin flows in cell migration: from locomotion and polarity to trajectories. Curr. Opin Cell Biol. 38, 12–17. (10.1016/j.ceb.2016.01.003) 26827283

[99] Théry M, Racine V, Piel M, Pepin A, Dimitrov A, Chen Y, Sibarita J-B, Bornens M 2006 Anisotropy of cell adhesive microenvironment governs cell internal organization and orientation of polarity. Proc. Natl Acad. Sci. USA 103, 19 771–19 776. (10.1073/pnas.0609267103) PMC1750916 17179050

[100] Balish MF 2014 Mycoplasma pneumoniae, an underutilized model for bacterial cell biology. J. Bacteriol. 196, 3675–3682. (10.1128/JB.01865-14) 25157081 PMC4248795

[101] Bloodgood RA 2010 Sensory reception is an attribute of both primary cilia and motile cilia. J. Cell Sci. 123, 505–509. (10.1242/jcs.066308) 20144998

[102] Delling M, DeCaen PG, Doerner JF, Febvay S, Clapham DE 2013 Primary cilia are specialized calcium signalling organelles. Nature 504, 311–314. (10.1038/nature12833) 24336288 PMC4112737

[103] DeCaen PG, Delling M, Vien TN, Clapham DE 2013 Direct recording and molecular identification of the calcium channel of primary cilia. Nature 504, 315–318. (10.1038/nature12832) 24336289 PMC4073646

[104] Nachury MV 2014 How do cilia organize signalling cascades? Phil. Trans. R. Soc. B 369, 20130465 (10.1098/rstb.2013.0465) 25047619 PMC4113109

[105] Bray D, Levin MD, Morton-Firth CJ 1998 Receptor clustering as a cellular mechanism to control sensitivity. Nature 393, 85–88. (10.1038/30018) 9590695

[106] Webre DJ, Wolanin PM, Stock JB 2003 Bacterial chemotaxis. Curr. Biol. 13, R47–R49. (10.1016/S0960-9822(02)01424-0) 12546801

[107] Shimizu TS, Aksenov SV, Bray D 2003 A spatially extended stochastic model of the bacterial chemotaxis signalling pathway. J. Mol. Biol. 329, 291–309. (10.1016/S0022-2836(03)00437-6) 12758077

[108] Tu Y 2013 Quantitative modeling of bacterial chemotaxis: signal amplification and accurate adaptation. Annu. Rev. Biophys. 42, 337–359. (10.1146/annurev-biophys-083012-130358) 23451887 PMC3737589

[109] Bray D 2015 Limits of computational biology. In Silico Biol. (Gedrukt). 12, 1–7. (10.3233/ISB-140461) 25318467 PMC4923711

[110] Bray D 2011 Wetware: a computer in every living cell. New Haven, CT: Yale University Press.

[111] Goldsmith TH 2013 Evolutionary tinkering with visual photoreception. Vis. Neurosci. 30, 21–37. (10.1017/S095252381200003X) 22391141

[112] Scherrington C 1963 Man on his nature. Cambridge, UK: Cambridge University Press.

[113] Beisson J et al. 2010 *Paramecium tetraurelia*: the renaissance of an early unicellular model. Cold. Spring. Harb. Protoc. 2010, pdb.emo140 (10.1101/pdb.emo140) 20150105

[114] Lwoff A 1950 Problems of morphogenesis in ciliates: *the kinetosomes in development, reproduction and evolution*. Yale J. Biol. Med. 23, 264.

[115] Beisson J, Sonneborn M 1965 Cytoplasmic inheritance of the organization of the cell cortex in *Paramecium aurelia*. Proc. Natl Acad. Sci. USA 53, 275–282. (10.1073/pnas.53.2.275) 14294056 PMC219507

[116] Bornens M 2008 Organelle positioning and cell polarity. Nat. Rev. Mol. Cell Biol. 9, 874–886. (10.1038/nrm2524) 18946476

[117] Li R, Gundersen GG 2008 Beyond polymer polarity: how the cytoskeleton builds a polarized cell. Nat. Rev. Mol. Cell Biol. 9, 860–873. (10.1038/nrm2522) 18946475

[118] Busiek KK, Margolin W 2015 Bacterial actin and tubulin homologs in cell growth and division. Curr. Biol. 25, R243–R254. (10.1016/j.cub.2015.01.030) 25784047 PMC5519336

[119] Gunning PW, Ghoshdastider U, Whitaker S, Popp D, Robinson RC 2015 The evolution of compositionally and functionally distinct actin filaments. J. Cell Sci. 128, 2009–2019. (10.1242/jcs.165563) 25788699

[120] Geuens G, Hill AM, Levilliers N, Adoutte A, DeBrabander M 1989 Microtubule dynamics investigated by microinjection of *Paramecium axonemal* tubulin: lack of nucleation but proximal assembly of microtubules at the kinetochore during prometaphase. J. Cell Biol. 108, 939–953. (10.1083/jcb.108.3.939) 2646309 PMC2115398

[121] Schuh M 2011 An actin-dependent mechanism for long-range vesicle transport. Nat. Cell Biol. 13, 1–7. (10.1038/ncb2353) 21983562 PMC3783939

[122] Lu W, Gelfand VI 2017 Moonlighting motors: kinesin, dynein, and cell polarity. Trends Cell Biol. 27, 505–514. (10.1016/j.tcb.2017.02.005) 28284467 PMC5476484

[123] Mitchison T, Kirschner M 1984 Dynamic instability of microtubule growth. Nature 312, 237–242. (10.1038/312237a0) 6504138

[124] Sale WS, Satir P 1976 Tetrahymena cilia: a system for analyzing sliding and axonemal spoke arrangements. J. Cell Biol. 71, 589–605. (10.1083/jcb.71.2.589) 825521 PMC2109766

[125] Xu Z et al. 2017 Microtubules acquire resistance from mechanical breakage through intralumenal acetylation. Science 356, 328–332. (10.1126/science.aai8764) 28428427 PMC5457157

[126] Portran D, Schaedel L, Xu Z, Théry M, Nachury MV 2017 Tubulin acetylation protects long-lived microtubules against mechanical ageing. Nat. Cell Biol. 19, 391–398. (10.1038/ncb3481) 28250419 PMC5376231

[127] Aumeier C, Schaedel L, Gaillard J, John K, Blanchoin L, Théry M 2016 Self-repair promotes microtubule rescue. Nat. Cell Biol. 18, 1054–1064. (10.1038/ncb3406) 27617929 PMC5045721

[128] Schaedel L, John K, Gaillard J, Nachury MV, Blanchoin L, Théry M 2015 Microtubules self-repair in response to mechanical stress. Nat. Mater. 14, 1156–1163. (10.1038/nmat4396) 26343914 PMC4620915

[129] Spang A et al. 2015 Complex archaea that bridge the gap between prokaryotes and eukaryotes. Nature 521, 173–179. (10.1038/nature14447) 25945739 PMC4444528

[130] Da Cunha V, Gaia M, Gadelle D, Nasir A, Forterre P 2017 Lokiarchaea are close relatives of Euryarchaeota, not bridging the gap between prokaryotes and eukaryotes. PLoS Genet. 13, e1006810 (10.1371/journal.pgen.1006810) 28604769 PMC5484517

[131] Koonin EV 2015 Energetics and population genetics at the root of eukaryotic cellular and genomic complexity. Proc. Natl Acad. Sci. USA 112, 15 777–15 778. (10.1073/pnas.1520869112) PMC4703004 26699503

[132] Zaremba-Niedzwiedzka K et al. 2017 Asgard archaea illuminate the origin of eukaryotic cellular complexity. Nature 541, 353–358. (10.1038/nature21031) 28077874

[133] Lewontin RC 1970 The units of selection. Annu. Rev. Ecol. Syst. 1, 1–18. (10.1146/annurev.es.01.110170.000245)

[134] Buss LW 1983 Evolution, development, and the units of selection. Proc. Natl Acad. Sci. USA 80, 1387–1391. (10.1073/pnas.80.5.1387) 6572396 PMC393602

[135] Michod RE 2007 Evolution of individuality during the transition from unicellular to multicellular life. Proc. Natl Acad. Sci. USA 104(Suppl. 1), 8613–8618. (10.1073/pnas.0701489104) 17494748 PMC1876437

[136] De Monte S, Rainey PB 2014 Nascent multicellular life and the emergence of individuality. J. Biosci. 39, 237–248. 24736157 10.1007/s12038-014-9420-5

[137] Szathmáry E 2015 Toward major evolutionary transitions theory 2.0. Proc. Natl Acad. Sci. USA 112, 10 104–10 111. (10.1073/pnas.1421398112) 25838283 PMC4547294

[138] Oberholzer M, Lopez MA, McLelland BT, Hill KL 2010 Social motility in African trypanosomes. PLoS Pathog. 6, e1000739 (10.1371/journal.ppat.1000739) 20126443 PMC2813273

[139] Saada EA, DeMarco SF, Shimogawa MM, Hill KL 2015 ‘With a little help from my friends'—social motility in *Trypanosoma brucei*. PLoS Pathog. 11, e1005272 (10.1371/journal.ppat.1005272) 26679190 PMC4683075

[140] Whiteley M, Diggle SP, Greenberg EP 2017 Progress in and promise of bacterial quorum sensing research. Nature 551, 313–320. (10.1038/nature24624) 29144467 PMC5870893

[141] Wolpert L, Szathmáry E 2002 Multicellularity: evolution and the egg. Nature 420, 745 (10.1038/420745a) 12490925

[142] King N 2004 The unicellular ancestry of animal development. Dev. Cell 7, 313–325. (10.1016/j.devcel.2004.08.010) 15363407

[143] Solari CA, Ganguly S, Kessler JO, Michod RE, Goldstein RE 2006 Multicellularity and the functional interdependence of motility and molecular transport. Proc. Natl Acad. Sci. USA 103, 1353–1358. (10.1073/pnas.0503810103) 16421211 PMC1360517

[144] Baskaran A, Marchetti MC 2009 Statistical mechanics and hydrodynamics of bacterial suspensions. Proc. Natl Acad. Sci. USA 106, 15 567–15 572. (10.1073/pnas.0906586106) PMC2747162 19717428

[145] Wickstead B, Gull K 2007 Dyneins across eukaryotes: a comparative genomic analysis. Traffic 8, 1708–1721. (10.1111/j.1600-0854.2007.00646.x) 17897317 PMC2239267

[146] Dettmer J, Friml J 2011 Cell polarity in plants: when two do the same, it is not the same. Curr. Opin. Cell Biol. 23, 686–696. (10.1016/j.ceb.2011.09.006) 21962973

[147] Lloyd C 2011 Dynamic microtubules and the texture of plant cell walls. Int. Rev. Cell Mol. Biol. 287, 287–329. (10.1016/B978-0-12-386043-9.00007-4) 21414591

[148] Wang D, Mills ES, Deal RB 2012 Technologies for systems-level analysis of specific cell types in plants. Plant Sci. 197, 21–29. (10.1016/j.plantsci.2012.08.012) 23116668 PMC4037754

[149] Kirk DL 2003 Seeking the ultimate and proximate causes of volvox multicellularity and cellular differentiation. Integr. Comp. Biol. 43, 247–253. (10.1093/icb/43.2.247) 21680429

[150] Hoops HJ 1997 Motility in the colonial and multicellular *Vovocales*: structure, function and evolution. Protoplasma 199, 99–112. (10.1007/BF01294499)

[151] Arakaki Y, Kawai-Toyooka H, Hamamura Y, Higashiyama T, Noga A, Hirono M, Olson BJSC, Nozaki H 2013 The simplest integrated multicellular organism unveiled. PLoS ONE 8, e81641 (10.1371/journal.pone.0081641) 24349103 PMC3859500

[152] James TY et al. 2006 Reconstructing the early evolution of Fungi using a six-gene phylogeny. Nature 443, 818–822. (10.1038/nature05110) 17051209

[153] McLaughlin DJ, Hibbett DS, Lutzoni F, Spatafora JW, Vilgalys R 2009 The search for the fungal tree of life. Trends Microbiol. 17, 488–497. (10.1016/j.tim.2009.08.001) 19782570

[154] McLaughlin DJ, Healy RA, Celio GJ, Roberson RW, Kumar TKA 2015 Evolution of zygomycetous spindle pole bodies: evidence from *Coemansia reversa* mitosis. Am. J. Bot. 102, 707–717. (10.3732/ajb.1400477) 26022485

[155] Kilmartin JV 2014 Lessons from yeast: the spindle pole body and the centrosome. Phil. Trans. R. Soc. B 369, 20130456, 10.1098/rstb.2013.0456. 25047610 PMC4113100

[156] Cavanaugh AM, Jaspersen SL 2017 Big lessons from little yeast: budding and fission yeast centrosome structure, duplication, and function. Annu. Rev. Genet. 51, 361–383. (10.1146/annurev-genet-120116-024733) 28934593

[157] Morris NR, Enos AP 1992 Mitotic gold in a mold: Aspergillus genetics and the biology of mitosis. Trends Genet. 8, 32–37. (10.1016/0168-9525(92)90022-V) 1369734

[158] Sasaki S, Shionoya A, Ishida M, Gambello MJ, Yingling J, Wynshaw-Boris A, Hirotsune S 2000 A LIS1/NUDEL/cytoplasmic dynein heavy chain complex in the developing and adult nervous system. Neuron 28, 681–696. (10.1016/S0896-6273(00)00146-X) 11163259

[159] Bornens M, Piel M 2002 Centrosome inheritance: birthright or the privilege of maturity? Curr. Biol. 12, R71–R73. (10.1016/S0960-9822(01)00678-9) 11818084

[160] Paoletti A, Bornens M 2003 Kar9 asymmetrical loading on spindle poles mediates proper spindle alignment in budding yeast. Dev. Cell 4, 289–290. (10.1016/S1534-5807(03)00065-0) 12636909

[161] Iskratsch T, Wolfenson H, Sheetz MP 2014 Appreciating force and shape—the rise of mechanotransduction in cell biology. Nat. Rev. Mol. Cell Biol. 15, 825–833. (10.1038/nrm3903) 25355507 PMC9339222

[162] Telford MJ, Budd GE, Philippe H 2015 Phylogenomic insights into animal evolution. Curr. Biol. 25, R876–R887. (10.1016/j.cub.2015.07.060) 26439351

[163] Arendt D et al. 2016 The origin and evolution of cell types. Nat. Rev. Genet. 17, 744–757. (10.1038/nrg.2016.127) 27818507

[164] Edelman GM 1988 Topobiology: an introduction to molecular embryology. New York, NY: Basic Books. 10.1126/science.243.4896.1373 17808269

[165] Abedin M, King N 2010 Diverse evolutionary paths to cell adhesion. Trends Cell Biol. 20, 734–742. (10.1016/j.tcb.2010.08.002) 20817460 PMC2991404

[166] Plusa B, Hadjantonakis A-K 2016 Mammalian development: mechanics drives cell differentiation. Nature 536, 281–282. (10.1038/nature18920) 27487214

[167] Friedl P, Mayor R 2017 Tuning collective cell migration by cell-cell junction regulation. Cold. Spring. Harb. Perspect. Biol. 9, pii:a029199 (10.1101/cshperspect.a029199) PMC5378050 28096261

[168] Frescas D, Mavrakis M, Lorenz H, Delotto R, Lippincott-Schwartz J 2006 The secretory membrane system in the *Drosophila* syncytial blastoderm embryo exists as functionally compartmentalized units around individual nuclei. J. Cell Biol. 173, 219–230. (10.1083/jcb.200601156) 16636144 PMC2063813

[169] Mavrakis M, Rikhy R, Lippincott-Schwartz J 2009 Plasma membrane polarity and compartmentalization are established before cellularization in the fly embryo. Dev. Cell 16, 93–104. (10.1016/j.devcel.2008.11.003) 19154721 PMC2684963

[170] Mavrakis M, Rikhy R, Lippincott-Schwartz J 2009 Cells within a cell: Insights into cellular architecture and polarization from the organization of the early fly embryo. Commun. Integr. Biol. 2, 313–314. (10.4161/cib.2.4.8240) 19721875 PMC2734032

[171] Freeman M, Nüsslein-Volhard C, Glover DM 1986 The dissociation of nuclear and centrosomal division in gnu, a mutation causing giant nuclei in Drosophila. Cell 46, 457–468. (10.1016/0092-8674(86)90666-5) 3089628

[172] Bornens M 2012 The centrosome in cells and organisms. Science 335, 422–426. (10.1126/science.1209037) 22282802

[173] Torruella G et al. 2015 Phylogenomics reveals convergent evolution of lifestyles in close relatives of animals and fungi. Curr. Biol. 25, 2404–2410. (10.1016/j.cub.2015.07.053) 26365255

[174] Mikhailov KV et al. 2009 The origin of Metazoa: a transition from temporal to spatial cell differentiation. Bioessays 31, 758–768. (10.1002/bies.200800214) 19472368

[175] Alegado RA, Brown LW, Cao S, Dermenjian RK, Zuzow R, Fairclough SR, Clardy J, King N 2012 A bacterial sulfonolipid triggers multicellular development in the closest living relatives of animals. Elife 1, e00013 (10.7554/eLife.00013) 23066504 PMC3463246

[176] Margulis L, Dolan MF, Guerrero R 2000 The chimeric eukaryote: origin of the nucleus from the karyomastigont in amitochondriate protists. Proc. Natl Acad. Sci. USA 97, 6954–6959. (10.1073/pnas.97.13.6954) 10860956 PMC34369

[177] Michod RE, Roze D 2001 Cooperation and conflict in the evolution of multicellularity. Heredity 86, 1–7. (10.1046/j.1365-2540.2001.00808.x) 11298810

[178] Kirkegaard JB, Goldstein RE 2016 Filter-feeding, near-field flows, and the morphologies of colonial choanoflagellates. Phys. Rev. E. 94, 052401 (10.1103/PhysRevE.94.052401) 27967109 PMC6054299

[179] Ludeman DA, Farrar N, Riesgo A, Paps J, Leys SP 2014 Evolutionary origins of sensation in metazoans: functional evidence for a new sensory organ in sponges. BMC Evol. Biol. 14, 3 (10.1186/1471-2148-14-3) 24410880 PMC3890488

[180] Heller E, Fuchs E 2015 Tissue patterning and cellular mechanics. J. Cell Biol. 211, 219–231. (10.1083/jcb.201506106) 26504164 PMC4621832

[181] Maître J-L et al. 2016 Asymmetric division of contractile domains couples cell positioning and fate specification. Nature 536, 344–348. (10.1038/nature18958) 27487217 PMC4998956

[182] Al Jord A et al. 2014 Centriole amplification by mother and daughter centrioles differs in multiciliated cells. Nature 516, 104–107. 25307055 10.1038/nature13770

[183] Rosenbaum JL, Witman GB 2002 Intraflagellar transport. Nat. Rev. Mol. Cell Biol. 3, 813–825. (10.1038/nrm952) 12415299

[184] Singla V, Reiter JF 2006 The primary cilium as the cell's antenna: signaling at a sensory organelle. Science 313, 629–633. (10.1126/science.1124534) 16888132

[185] Satir P, Christensen ST 2007 Overview of structure and function of mammalian cilia. Annu. Rev. Physiol. 69, 377–400. (10.1146/annurev.physiol.69.040705.141236) 17009929

[186] Gerdes JM, Davis EE, Katsanis N 2009 The vertebrate primary cilium in development, homeostasis, and disease. Cell 137, 32–45. (10.1016/j.cell.2009.03.023) 19345185 PMC3016012

[187] Goetz SC, Anderson KV 2010 The primary cilium: a signalling centre during vertebrate development. Nat. Rev. Genet. 11, 331–344. (10.1038/nrg2774) 20395968 PMC3121168

[188] Farnum CE, Wilsman NJ 2011 Axonemal positioning and orientation in three-dimensional space for primary cilia: what is known, what is assumed, and what needs clarification. Dev. Dyn. 240, 2405–2431. (10.1002/dvdy.22756) 22012592 PMC3278774

[189] Mahjoub MR 2013 The importance of a single primary cilium. Organogenesis 9, 61–69. (10.4161/org.25144) 23819944 PMC3812286

[190] Izawa I, Goto H, Kasahara K, Inagaki M 2015 Current topics of functional links between primary cilia and cell cycle. Cilia 4, 12 (10.1186/s13630-015-0021-1) 26719793 PMC4696186

[191] Braun DA, Hildebrandt F 2017 Ciliopathies. Cold. Spring. Harb. Perspect. Biol. 9, a028191 (10.1101/cshperspect.a028191) 27793968 PMC5334254

[192] Hsu K-S, Chuang J-Z, Sung C-H 2017 The biology of ciliary dynamics. Cold. Spring. Harb. Perspect. Biol. 9, a027904 (10.1101/cshperspect.a027904) 28062565 PMC5378047

[193] He M, Agbu S, Anderson KV 2017 Microtubule motors drive hedgehog signaling in primary cilia. Trends Cell Biol. 27, 110–125. (10.1016/j.tcb.2016.09.010) 27765513 PMC5258846

[194] Malicki JJ, Johnson CA 2017 The cilium: cellular antenna and central processing unit. Trends Cell Biol. 27, 126–140. (10.1016/j.tcb.2016.08.002) 27634431 PMC5278183

[195] Winey M, O'Toole E 2014 Centriole structure. Phil. Trans. R. Soc. B 369, 20130457, 10.1098/rstb.2013.0457. 25047611 PMC4113101

[196] Doxsey S, McCollum D, Theurkauf W 2005 Centrosomes in cellular regulation. Annu. Rev. Cell Dev. Biol. 21, 411–434. (10.1146/annurev.cellbio.21.122303.120418) 16212501

[197] Badano JL, Teslovich TM, Katsanis N 2005 The centrosome in human genetic disease. Nat. Rev. Genet. 6, 194–205. (10.1038/nrg1557) 15738963

[198] Tsou M-FB, Stearns T 2006 Controlling centrosome number: licenses and blocks. Curr. Opin Cell Biol. 18, 74–78. (10.1016/j.ceb.2005.12.008) 16361091

[199] Nigg EA, Raff JW 2009 Centrioles, centrosomes, and cilia in health and disease. Cell 139, 663–678. (10.1016/j.cell.2009.10.036) 19914163

[200] Sillibourne JE, Bornens M 2010 Polo-like kinase 4: the odd one out of the family. Cell Div. 5, 25 (10.1186/1747-1028-5-25) 20920249 PMC2955731

[201] Azimzadeh J, Marshall WF 2010 Building the centriole. Curr. Biol. 20, R816–R825. (10.1016/j.cub.2010.08.010) 20869612 PMC2956124

[202] Nigg EA, Stearns T 2011 The centrosome cycle: centriole biogenesis, duplication and inherent asymmetries. Nat. Cell Biol. 13, 1154–1160. (10.1038/ncb2345) 21968988 PMC3947860

[203] Gönczy P 2012 Towards a molecular architecture of centriole assembly. Nat. Rev. Mol. Cell Biol. 13, 425–435. (10.1038/nrm3373) 22691849

[204] Mardin BR, Schiebel E 2012 Breaking the ties that bind: new advances in centrosome biology. J. Cell Biol. 197, 11–18. (10.1083/jcb.201108006) 22472437 PMC3317805

[205] Chavali PL, Pütz M, Gergely F 2014 Small organelle, big responsibility: the role of centrosomes in development and disease. Phil. Trans. R. Soc. B 369, 20130468 (10.1098/rstb.2013.0468) 25047622 PMC4113112

[206] Arquint C, Gabryjonczyk A.-M, Nigg EA 2014 Centrosomes as signalling centres. Phil. Trans. R. Soc. B 369, 20130464 (10.1098/rstb.2013.0464) 25047618 PMC4113108

[207] Woodruff JB, Wueseke O, Hyman AA 2014 Pericentriolar material structure and dynamics. Phil. Trans. R. Soc. B 369, 20130459 (10.1098/rstb.2013.0459) 25047613 PMC4113103

[208] Sluder G 2014 One to only two: a short history of the centrosome and its duplication. Phil. Trans. R. Soc. B 369, 20130455 (10.1098/rstb.2013.0455) 25047609 PMC4113099

[209] Conduit PT, Wainman A, Raff JW 2015 Centrosome function and assembly in animal cells. Nat. Rev. Mol. Cell Biol. 16, 611–624. (10.1038/nrm4062) 26373263

[210] Gönczy P 2015 Centrosomes and cancer: revisiting a long-standing relationship. Nat. Rev. Cancer 15, 639–652. (10.1038/nrc3995) 26493645

[211] Vertii A, Hehnly H, Doxsey S 2016 The centrosome, a multitalented renaissance organelle. Cold. Spring. Harb. Perspect. Biol. 8, pii:a025049 (10.1101/cshperspect.a025049) PMC5131770 27908937

[212] Nano M, Basto R 2016 The Janus soul of centrosomes: a paradoxical role in disease? Chromosome Res. 24, 127–144. (10.1007/s10577-015-9507-3) 26643310

[213] Lattao R, Kovács L, Glover DM 2017 The centrioles, centrosomes, basal bodies, and cilia of *Drosophila melanogaster*. Genetics 206, 33–53. (10.1534/genetics.116.198168) 28476861 PMC5419478

[214] Paz J, Lüders J 2017 Microtubule-organizing centers: towards a minimal parts list. Trends Cell Biol. 28, 176–187. (10.1016/j.tcb.2017.10.005) 29173799

[215] Bloodgood RA 2009 From central to rudimentary to primary: the history of an underappreciated organelle whose time has come. The primary cilium. Methods Cell Biol. 94, 3–52. 20362083 10.1016/S0091-679X(08)94001-2

[216] Gupta GD et al. 2015 A dynamic protein interaction landscape of the human centrosome–cilium interface. Cell 163, 1484–1499. (10.1016/j.cell.2015.10.065) 26638075 PMC5089374

[217] Tateishi K, Yamazaki Y, Nishida T, Watanabe S, Kunimoto K, Ishikawa H, Tsukita S 2013 Two appendages homologous between basal bodies and centrioles are formed using distinct Odf2 domains. J. Cell Biol. 203, 417–425. (10.1083/jcb.201303071) 24189274 PMC3824012

[218] Bornens M, Gönczy P 2014 Centrosomes back in the limelight. Phil. Trans. R. Soc. B 369, 20130452, 10.1098/rstb.2013.0452. 25047606 PMC4113096

[219] Debec A, Sullivan W, Bettencourt-Dias M 2010 Centrioles: active players or passengers during mitosis? Cell. Mol. Life Sci. 67, 2173–2194. (10.1007/s00018-010-0323-9) 20300952 PMC2883084

[220] Chavali PL, Peset I, Gergely F 2015 Centrosomes and mitotic spindle poles: a recent liaison? Biochem. Soc. Trans 43, 13–18. (10.1042/BST20140269) 25619241

[221] Carvalho-Santos Z, Machado P, Branco P, Tavares-Cadete F, Rodrigues-Martins A, Pereira-Leal JB, Bettencourt-Dias M 2010 Stepwise evolution of the centriole-assembly pathway. J. Cell Sci. 123, 1414–1426. (10.1242/jcs.064931) 20392737

[222] Hodges ME, Scheumann N, Wickstead B, Langdale JA, Gull K 2010 Reconstructing the evolutionary history of the centriole from protein components. J. Cell Sci. 123, 1407–1413. (10.1242/jcs.064873) 20388734 PMC2858018

[223] Carvalho-Santos Z, Azimzadeh J, Pereira-Leal JB, Bettencourt-Dias M 2011 Evolution: tracing the origins of centrioles, cilia, and flagella. J. Cell Biol. 194, 165–175. (10.1083/jcb.201011152) 21788366 PMC3144413

[224] Sir J-H, Daly O, Morrison CG, Dunning M, Kilmartin JV, Gergely F 2013 Loss of centrioles causes chromosomal instability in vertebrate somatic cells. J. Cell Biol. 203, 747–756. (10.1083/jcb.201309038) 24297747 PMC3857480

[225] Bazzi H, Anderson KV 2014 Acentriolar mitosis activates a p53-dependent apoptosis pathway in the mouse embryo. Proc. Natl Acad. Sci. USA 111, E1491–E1500. (10.1073/pnas.1400568111) 24706806 PMC3992648

[226] Bailly E, Bornens M 1992 Cell biology. Centrosome and cell division. Nature 355, 300–301. (10.1038/355300a0) 1731242

[227] Pines J, Hagan I 2011 The Renaissance or the cuckoo clock. Phil. Trans. R. Soc. B 366, 3625–3634. (10.1098/rstb.2011.0080) 22084388 PMC3203460

[228] Al Jord A et al. 2017 Calibrated mitotic oscillator drives motile ciliogenesis. Science 358, 803–806. (10.1126/science.aan8311) 28982797

[229] Chan KY et al. 2017 Dialogue between centrosomal entrance and exit scaffold pathways regulates mitotic commitment. J. Cell Biol. 216, 2795–2812. 28774892 10.1083/jcb.201702172 PMC5584178

[230] Meitinger F et al. 2016 53BP1 and USP28 mediate p53 activation and G1 arrest after centrosome loss or extended mitotic duration. J. Cell Biol. 214, 155–166. (10.1083/jcb.201604081) 27432897 PMC4949453

[231] Piel M, Meyer P, Khodjakov A, Rieder CL, Bornens M 2000 The respective contributions of the mother and daughter centrioles to centrosome activity and behavior in vertebrate cells. J. Cell Biol. 149, 317–330. (10.1083/jcb.149.2.317) 10769025 PMC2175166

[232] Piel M, Nordberg J, Euteneuer U, Bornens M 2001 Centrosome-dependent exit of cytokinesis in animal cells. Science 291, 1550–1553. (10.1126/science.1057330) 11222861

[233] Suvorova ES, Francia M, Striepen B, White MW 2015 A novel bipartite centrosome coordinates the apicomplexan cell cycle. PLoS Biol. 13, e1002093 (10.1371/journal.pbio.1002093) 25734885 PMC4348508

[234] Ishihara K, Nguyen PA, Wuhr M, Groen AC, Field CM, Mitchison TJ 2014 Organization of early frog embryos by chemical waves emanating from centrosomes. Phil. Trans. R. Soc. B 369, 20130454 (10.1098/rstb.2013.0454) 25047608 PMC4113098

[235] Tournier F, Bornens M 2001 Centrosomes and parthenogenesis. Methods Cell Biol. 67, 213–224. (10.1016/S0091-679X(01)67015-8) 11550470

[236] Terrin A et al. 2012 PKA and PDE4D3 anchoring to AKAP9 provides distinct regulation of cAMP signals at the centrosome. J. Cell Biol. 198, 607–621. (10.1083/jcb.201201059) 22908311 PMC3514031

[237] Farina F et al. 2016 The centrosome is an actin-organizing centre. Nat. Cell Biol. 18, 65–75. (10.1038/ncb3285) 26655833 PMC4880044

[238] Obino D et al. 2016 Actin nucleation at the centrosome controls lymphocyte polarity. Nat Commun. 7, 10969 (10.1038/ncomms10969) 26987298 PMC4802043

[239] Hehnly H, Chen C-T, Powers CM, Liu H-L, Doxsey S 2012 The centrosome regulates the Rab11- dependent recycling endosome pathway at appendages of the mother centriole. Curr. Biol. 22, 1944–1950. (10.1016/j.cub.2012.08.022) 22981775 PMC3917512

[240] Gräf R, Euteneuer U, Ho T-H, Rehberg M 2003 Regulated expression of the centrosomal protein DdCP224 affects microtubule dynamics and reveals mechanisms for the control of supernumerary centrosome number. Mol. Biol. Cell 14, 4067–4074. (10.1091/mbc.e03-04-0242) 14517319 PMC207000

[241] Minc N, Burgess D, Chang F 2011 Influence of cell geometry on division-plane positioning. Cell 144, 414–426. (10.1016/j.cell.2011.01.016) 21295701 PMC3048034

[242] Behrens R, Nurse P 2002 Roles of fission yeast tea1p in the localization of polarity factors and in organizing the microtubular cytoskeleton. J. Cell Biol. 157, 783–793. (10.1083/jcb.200112027) 12034771 PMC2173414

[243] Gottardo M, Callaini G, Riparbelli MG 2015 The *Drosophila* centriole: conversion of doublets into triplets within the stem cell niche. J. Cell Sci. 128, 2437–2442. (10.1242/jcs.172627) 26092937

[244] Li S, Sandercock AM, Conduit P, Robinson CV, Williams RL, Kilmartin JV 2006 Structural role of Sfi1p-centrin filaments in budding yeast spindle pole body duplication. J. Cell Biol. 173, 867–877. (10.1083/jcb.200603153) 16785321 PMC2063913

[245] Bouhlel IB et al. 2015 Cell cycle control of spindle pole body duplication and splitting by Sfi1 and Cdc31 in fission yeast. J. Cell Sci. 128, 1481–1493. (10.1242/jcs.159657) 25736294

[246] Tournier F, Bobinnec Y, Debec A, Santamaria P, Bornens M 1999 *Drosophila* centrosomes are unable to trigger parthenogenetic development of Xenopus eggs. Biol. Cell 91, 99–108. (10.1016/S0248-4900(99)80034-3) 10399825

[247] Rodrigues-Martins A, Riparbelli M, Callaini G, Glover DM, Bettencourt-Dias M 2008 From centriole biogenesis to cellular function: centrioles are essential for cell division at critical developmental stages. Cell Cycle 7, 11–16. (10.4161/cc.7.1.5226) 18196975

[248] Megraw TL, Kao LR, Kaufman TC 2001 Zygotic development without functional mitotic centrosomes. Curr. Biol. 11, 116–120. (10.1016/S0960-9822(01)00017-3) 11231128

[249] Basto R, Lau J, Vinogradova T, Gardiol A, Woods CG, Khodjakov A, Raff JW 2006 Flies without centrioles. Cell 125, 1375–1386. (10.1016/j.cell.2006.05.025) 16814722

[250] Poulton JS, Cuningham JC, Peifer M 2014 Acentrosomal *Drosophila* epithelial cells exhibit abnormal cell division, leading to cell death and compensatory proliferation. Dev. Cell 30, 731–745. (10.1016/j.devcel.2014.08.007) 25241934 PMC4182331

[251] Dallai R, Lupetti P, Mencarelli C 2006 Unusual axonemes of hexapod spermatozoa. Int. Rev. Cytol. 254, 45–99. (10.1016/S0074-7696(06)54002-1) 17147997

[252] Ross L, Normark BB 2015 Evolutionary problems in centrosome and centriole biology. J. Evol. Biol. 28, 995–1004. (10.1111/jeb.12620) 25781035 PMC4979663

[253] Wilkin MB et al. 2000 *Drosophila* dumpy is a gigantic extracellular protein required to maintain tension at epidermal-cuticle attachment sites. Curr. Biol. 10, 559–567. (10.1016/S0960-9822(00)00482-6) 10837220

[254] Abal M, Keryer G, Bornens M 2005 Centrioles resist forces applied on centrosomes during G2/M transition. Biol. Cell 97, 425–434. (10.1042/BC20040112) 15898952

[255] Sanders WM, Kaverina I 2015 Nucleation and dynamics of golgi-derived microtubules. Front Neurosci. 9, 431 (10.3389/fnins.2015.00431) 26617483 PMC4639703

[256] Rivero S, Cardenas J, Bornens M, Rios RM 2009 Microtubule nucleation at the cis-side of the Golgi apparatus requires AKAP450 and GM130. EMBO J. 28, 1016–1028. (10.1038/emboj.2009.47) 19242490 PMC2683699

[257] Yang R, Feldman JL 2015 SPD-2/CEP192 and CDK are limiting for microtubule-organizing center function at the centrosome. Curr. Biol. 25, 1924–1931. (10.1016/j.cub.2015.06.001) 26119750

[258] Muroyama A, Lechler T 2017 Microtubule organization, dynamics and functions in differentiated cells. Development 144, 3012–3021. (10.1242/dev.153171) 28851722 PMC5611961

[259] Delgehyr N, Sillibourne J, Bornens M 2005 Microtubule nucleation and anchoring at the centrosome are independent processes linked by ninein function. J. Cell Sci. 118, 1565–1575. (10.1242/jcs.02302) 15784680

[260] Goldspink DA et al. 2017 Ninein is essential for apico-basal microtubule formation and CLIP-170 facilitates its redeployment to non-centrosomal microtubule organizing centres. Open Biol. 7, 160274 (10.1098/rsob.160274) 28179500 PMC5356440

[261] Gavilan MP, Arjona M, Zurbano A, Formstecher E, Martinez-Morales JR, Bornens M, Rios RM 2015 Alpha-catenin-dependent recruitment of the centrosomal protein CAP350 to adherens junctions allows epithelial cells to acquire a columnar shape. PLoS Biol. 13, e1002087 (10.1371/journal.pbio.1002087) 25764135 PMC4357431

[262] Stinchcombe JC, Majorovits E, Bossi G, Fuller S, Griffiths GM 2006 Centrosome polarization delivers secretory granules to the immunological synapse. Nature 443, 462–465. (10.1038/nature05071) 17006514

[263] Laan L, Roth S, Dogterom M 2012 End-on microtubule-dynein interactions and pulling-based positioning of microtubule organizing centers. Cell Cycle 11, 3750–3757. (10.4161/cc.21753) 22895049 PMC3495818

[264] Chevrier V, Piel M, Collomb N, Saoudi Y, Frank R, Paintrand M, Narumiya S, Bornens M, Job D 2002 The Rho-associated protein kinase p160ROCK is required for centrosome positioning. J. Cell Biol. 157, 807–817. (10.1083/jcb.200203034) 12034773 PMC2173415

[265] Klotz C, Bordes N, Laine MC, Sandoz D, Bornens M 1986 Myosin at the apical pole of ciliated epithelial cells as revealed by a monoclonal antibody. J. Cell Biol. 103, 613–619. (10.1083/jcb.103.2.613) 3525577 PMC2113832

[266] Antoniades I, Stylianou P, Skourides PA 2014 Making the connection: ciliary adhesion complexes anchor basal bodies to the actin cytoskeleton. Dev. Cell 28, 70–80. (10.1016/j.devcel.2013.12.003) 24434137

[267] Tassin AM, Maro B, Bornens M 1985 Fate of microtubule-organizing centers during myogenesis *in vitro*. J. Cell Biol. 100, 35–46. (10.1083/jcb.100.1.35) 3880758 PMC2113478

[268] Lambert JD, Nagy LM 2002 Asymmetric inheritance of centrosomally localized mRNAs during embryonic cleavages. Nature 420, 682–686. (10.1038/nature01241) 12478296

[269] Tozer S, Baek C, Fischer E, Goiame R, Morin X 2017 Differential routing of mindbomb1 via centriolar satellites regulates asymmetric divisions of neural progenitors. Neuron 93, 542–551. (10.1016/j.neuron.2016.12.042) 28132826

[270] Extavour CG, Akam M 2003 Mechanisms of germ cell specification across the metazoans: epigenesis and preformation. Development 130, 5869–5884. (10.1242/dev.00804) 14597570

[271] Swartz SZ, Wessel GM 2015 Germ line versus soma in the transition from egg to embryo. Curr. Top. Dev. Biol. 113, 149–190. (10.1016/bs.ctdb.2015.06.003) 26358873 PMC5094369

[272] Mello CC, Schubert C, Draper B, Zhang W, Lobel R, Priess JR 1996 The PIE-1 protein and germline specification in *C. elegans* embryos. Nature 382, 710–712. (10.1038/382710a0) 8751440

[273] Lerit DA, Gavis ER 2011 Transport of germ plasm on astral microtubules directs germ cell development in *Drosophila*. Curr. Biol. 21, 439–448. (10.1016/j.cub.2011.01.073) 21376599 PMC3062663

[274] Raff JW, Glover DM 1989 Centrosomes, and not nuclei, initiate pole cell formation in *Drosophila* embryos. Cell 57, 611–619. (10.1016/0092-8674(89)90130-X) 2497990

[275] Pepling ME, Wilhelm JE, O'Hara AL, Gephardt GW, Spradling AC 2007 Mouse oocytes within germ cell cysts and primordial follicles contain a Balbiani body. Proc. Natl Acad. Sci. USA 104, 187–192. (10.1073/pnas.0609923104) 17189423 PMC1765432

[276] Boke E et al. 2016 Amyloid-like self-assembly of a cellular compartment. Cell 166, 637–650. (10.1016/j.cell.2016.06.051) 27471966 PMC5082712

[277] Elkouby YM, Jamieson-Lucy A, Mullins MC 2016 Oocyte polarization is coupled to the chromosomal bouquet, a conserved polarized nuclear configuration in meiosis. PLoS Biol. 14, e1002335 (10.1371/journal.pbio.1002335) 26741740 PMC4704784

[278] Elkouby YM, Mullins MC 2017 Coordination of cellular differentiation, polarity, mitosis and meiosis: new findings from early vertebrate oogenesis. Dev. Biol. 430, 275–287. (10.1016/j.ydbio.2017.06.029) 28666956 PMC5623617

[279] Elkouby YM 2017 All in one: integrating cell polarity, meiosis, mitosis and mechanical forces in early oocyte differentiation in vertebrates. Int. J. Dev. Biol. 61, 179–193. (10.1387/ijdb.170030ye) 28621416

[280] Inoue D, Wittbrodt J, Gruss OJ 2018 Loss and rebirth of the animal microtubule organizing center: how maternal expression of centrosomal proteins cooperates with the sperm centriole in zygotic centrosome reformation. Bioessays 40, e1700135 (10.1002/bies.201700135) 29522658

[281] Christophorou N, Rubin T, Bonnet I, Piolot T, Arnaud M 2015 Microtubule-driven nuclear rotations promote meiotic chromosome dynamics. Nat. Cell Biol. 17, 1388–1400. (10.1038/ncb3249) 26458247

[282] Anderson JK, Smith TG, Hoover TR 2010 Sense and sensibility: flagellum-mediated gene regulation. Trends Microbiol. 18, 30–37. (10.1016/j.tim.2009.11.001) 19942438 PMC2818477

[283] Paluch EK et al. 2015 Mechanotransduction: use the force(s). BMC Biol. 13, 47 (10.1186/s12915-015-0150-4) 26141078 PMC4491211

[284] Saló E, Baguñà J 2002 Regeneration in planarians and other worms: new findings, new tools, and new perspectives. J. Exp. Zool. 292, 528–539. (10.1002/jez.90001) 12115936

[285] Bely AE 2006 Distribution of segment regeneration ability in the Annelida. Integr. Comp. Biol. 46, 508–518. (10.1093/icb/icj051) 21672762

[286] Egger B, Gschwentner R, Rieger R 2007 Free-living flatworms under the knife: past and present. Dev. Genes Evol. 217, 89–104. (10.1007/s00427-006-0120-5) 17146688 PMC1784541

[287] Egger B 2008 Regeneration: rewarding, but potentially risky. Birth Defects Res. C Embryo Today 84, 257–264. (10.1002/bdrc.20135) 19067421

[288] Newmark PA, Sánchez Alvarado A 2002 Not your father's planarian: a classic model enters the era of functional genomics. Nat. Rev. Genet. 3, 210–219. (10.1038/nrg759) 11972158

[289] Wagner DE, Wang IE, Reddien PW 2011 Clonogenic neoblasts are pluripotent adult stem cells that underlie planarian regeneration. Science 332, 811–816. (10.1126/science.1203983) 21566185 PMC3338249

[290] Grohme MA et al. 2018 The genome of *Schmidtea mediterranea* and the evolution of core cellular mechanisms. Nature 554, 56–61. (10.1038/nature25473) 29364871 PMC5797480

[291] Azimzadeh J, Wong ML, Downhour DM, Sánchez Alvarado A, Marshall WF 2012 Centrosome loss in the evolution of planarians. Science 335, 461–463. (10.1126/science.1214457) 22223737 PMC3347778

[292] Petersen CP, Reddien PW 2009 Wnt signaling and the polarity of the primary body axis. Cell 139, 1056–1068. (10.1016/j.cell.2009.11.035) 20005801

[293] Mbom BC, Nelson WJ, Barth A 2013 β-catenin at the centrosome: discrete pools of β-catenin communicate during mitosis and may co-ordinate centrosome functions and cell cycle progression. Bioessays 35, 804–809. (10.1002/bies.201300045) 23804296 PMC3983869

[294] Habib SJ, Chen B-C, Tsai F-C, Anastassiadis K, Meyer T, Betzig E, Nusse R 2013 A localized Wnt signal orients asymmetric stem cell division in vitro. Science 339, 1445–1448. (10.1126/science.1231077) 23520113 PMC3966430

[295] Lapébie P, Borchiellini C, Houliston E 2011 Dissecting the PCP pathway: one or more pathways? Does a separate Wnt-Fz-Rho pathway drive morphogenesis? Bioessays 33, 759–768. (10.1002/bies.201100023) 21919026

[296] Momose T, Kraus Y, Houliston E 2012 A conserved function for Strabismus in establishing planar cell polarity in the ciliated ectoderm during cnidarian larval development. Development 139, 4374–4382. (10.1242/dev.084251) 23095884

[297] Richards E 2017 Darwin and the making of sexual selection. Chicago, IL: University of Chicago Press.

[298] Jones AG, Ratterman NL 2009 Mate choice and sexual selection: what have we learned since Darwin? Proc. Natl Acad. Sci. USA 106(Suppl. 1), 10 001–10 008. (10.1073/pnas.0901129106) 19528643 PMC2702796

[299] Smith JM 1991 Theories of sexual selection. Trends Ecol. Evol. 6, 146–151. (10.1016/0169-5347(91)90055-3) 21232444

[300] Wang X, Tsai J-W, Imai JH, Lian W-N, Vallee RB, Shi S-H 2009 Asymmetric centrosome inheritance maintains neural progenitors in the neocortex. Nature 461, 947–955. (10.1038/nature08435) 19829375 PMC2764320

[301] Wilsch-Bräuninger M, Peters J, Paridaen ML, Huttner WB 2012 Basolateral rather than apical primary cilia on neuroepithelial cells committed to delamination. Development 139, 95–105. (10.1242/dev.069294) 22096071

[302] Jékely G 2011 Origin and early evolution of neural circuits for the control of ciliary locomotion. Proc. R. Soc. B 278, 914–922. (10.1098/rspb.2010.2027) PMC3049052 21123265

[303] Gilpin W, Prakash VN, Prakash M 2017 Vortex arrays and ciliary tangles underlie the feeding-swimming trade-off in starfish karvae. Nat. Phys. 13, 380–386. (10.1038/nphys3981)

[304] Jékely G, Arendt D 2006 Evolution of intraflagellar transport from coated vesicles and autogenous origin of the eukaryotic cilium. Bioessays 28, 191–198. (10.1002/bies.20369) 16435301

[305] Lane N, Martin W 2010 The energetics of genome complexity. Nature 467, 929–934. (10.1038/nature09486) 20962839

[306] Loncarek J, Bettencourt-Dias M 2018 Building the right centriole for each cell type. J. Cell Biol. 217, 823–835. (10.1083/jcb.201704093) 29284667 PMC5839779

